# Benchmarking of Quantitative Proteomics Workflows for Limited Proteolysis Mass Spectrometry

**DOI:** 10.1016/j.mcpro.2025.100945

**Published:** 2025-03-13

**Authors:** Tomas Koudelka, Claudio Bassot, Ilaria Piazza

**Affiliations:** 1 Max-Delbrück-Center for Molecular Medicine in the Helmholtz Association (MDC), Berlin, Germany; 2 SciLifeLab, Department of Microbiology, Tumor and Cell Biology, Karolinska Institutet, Solna, Sweden

**Keywords:** LiP-MS, DIA-MS, TMT, FAIMS, structural proteomics, DIA software benchmarking, FragPipe

## Abstract

Limited proteolysis coupled with mass spectrometry (LiP-MS) has emerged as a powerful technique for detecting protein structural changes and drug-protein interactions on a proteome-wide scale. However, there is no consensus on the best quantitative proteomics workflow for analyzing LiP-MS data. In this study, we comprehensively benchmarked two major quantification approaches—data-independent acquisition (DIA) and tandem mass tag (TMT) isobaric labeling—in combination with LiP-MS, using a drug-target deconvolution assay as a model system. Our results show that while TMT labeling enabled the quantification of more peptides and proteins with lower coefficients of variation, DIA-MS exhibited greater accuracy in identifying true drug targets and stronger dose-response correlation in peptides of protein targets. Additionally, we evaluated the performance of freely available (FragPipe) versus commercial (Spectronaut) software tools for DIA-MS analysis, revealing that the choice between precision (FragPipe) and sensitivity (Spectronaut) largely depends on the specific experimental context. Our findings underscore the importance of selecting the appropriate LiP-MS quantification strategy based on the study objectives. This work provides valuable guidelines for researchers in structural proteomics and drug discovery, and highlights how advancements in mass spectrometry instrumentation, such as the Astral mass spectrometer, may further improve sensitivity and protein sequence coverage, potentially reducing the need for TMT labeling.

## Highlights

• LiP-MS experiments face unique challenges due to peptide-level quantification.
• Benchmarking 12 workflows to evaluate LiP-MS for protein structural change detection.
• Both DIA and TMT are effective LiP-MS quantification strategies.
• Open-source tools (FragPipe/DIA-NN) provide robust peptide quantification for LiP-MS.
• Experiment-specific spectral libraries enhance DIA-MS sensitivity.

## Graphical Abstract

**Figure:**
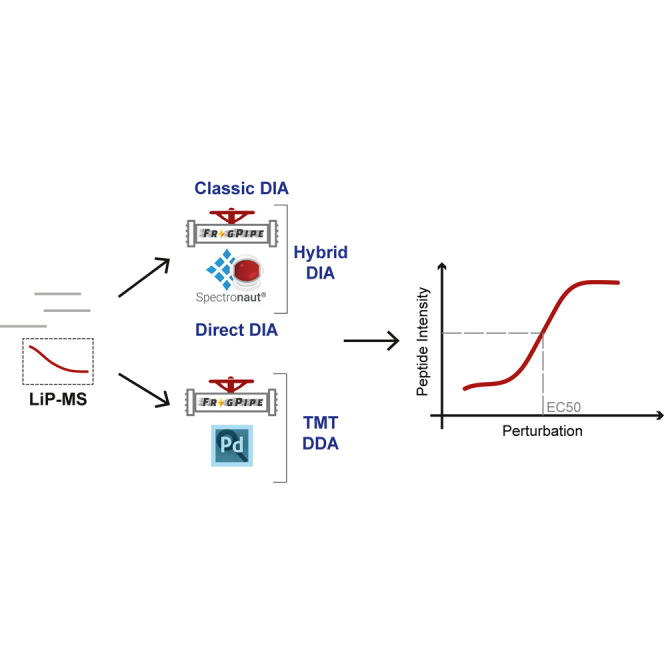

## In Brief

We evaluate different mass spectrometry-based workflows for quantifying protein structural changes using limited proteolysis coupled with mass spectrometry (LiP-MS), a technique of increasing relevance in structural proteomics and drug discovery. In this study, we compared the performance of data-independent acquisition and tandem mass tag isobaric labeling approaches, with a focus on their effectiveness in detecting protein structural changes by LiP-MS. We offer insights into the strengths and limitations of data acquisition and analysis software for LiP-MS quantification.

Limited proteolysis coupled with mass spectrometry (LiP-MS) is a proteomics technique designed to detect protein structural changes across an entire proteome (1, 2, 3). Recognized as one of the most robust methods for direct structural biology analysis in complex biological systems, LiP-MS involves a controlled proteolytic digestion of cellular lysates using a broad-specificity protease. This process creates cleavage sites that reflect the structural states of proteins. LiP-MS has proven effective in studying protein structural changes in response to environmental stress, as well as in analyzing protein-protein interactions and protein aggregation (4). In the context of drug discovery, LiP-MS is particularly valuable for identifying drug targets across various compound classes and species, including microbes and human cells or tissues (5, 6, 7, 8). LiP-MS facilitates the unbiased discovery of drug targets among thousands of proteins using bottom-up mass spectrometry. The relative abundance of peptides generated after limited proteolysis serves as the quantitative measure of protein structural changes, making accurate peptide quantification by mass spectrometry crucial. Currently, no standardized quantitative proteomics strategy exists for achieving the best results in LiP-MS. Early studies employed selected reaction monitoring (SRM) and data-dependent acquisition (DDA) workflows for peptide quantification. Recently, data-independent acquisition (DIA) mass spectrometry has emerged as a reliable alternative (9), offering broader proteomic coverage, consistent and accurate quantification of limited proteolysis features, and reduced missing values in the data.

Despite these advantages, DIA-MS presents challenges in LiP-MS data processing, due to the complex mass spectra generated by the coisolation and cofragmentation of multiple precursors within the same selection window. This complexity is further compounded by the use of semispecific searches in LiP-MS, which can increase the search space by approximately 20 times the expected number of tryptic peptides. The computational analysis of DIA data typically involves two major steps: creating a targeted spectral library and extracting quantification features for all peptide ions. Search engine databases like MSFragger-DIA (10), DIA-Umpire (11), and Pulsar (12) are commonly used for peptide identification, while DIA-NN (13) and Spectronaut (14) are software tools employed for targeted extraction. The choice of software tools for data analysis and the design of spectral libraries significantly impact the outcomes of DIA-MS in global proteomics and phosphoproteomics studies (15, 16). However, the experimental design and DIA data analysis strategies have not yet been properly benchmarked for limited proteolysis data.

LiP-MS measures protein structural alterations at the peptide sequence level, which in drug deconvolution experiments, allows for the identification of candidate protein targets through changes in peptide abundance upon drug binding (referred to as LiP-peptides). Moreover, the specific positions of these LiP-peptides can provide structural insights at the protein domain level, including potential drug binding sites. However, this method has its limitations. While the data richness from LiP-MS is significant, some protein-drug interactions or conformational changes might remain undetected due to incomplete protein sequence coverage. Isobaric labeling-based quantification with tandem mass tag (TMT) offers a different strategy that could potentially enhance the depth of analysis and reduce missing values compared to DIA or, in general, label-free approaches. For example, Ruwolt *et al*. demonstrated that TMT labeling improves overall quantitative performance in proteome-wide cross-linking mass spectrometry (17). However, it is still unclear whether the benefits of TMT can be fully realized in LiP-MS, as the potential issue of ratio compression might negatively impact peptide quantification quality.

A consensus has yet to be reached on the optimal software and quantitative proteomics experimental design, such as isobaric labeling versus label-free approaches, for processing limited proteolysis data. The impact of these choices on LiP-MS assay outcomes has not been thoroughly investigated, particularly using benchmark data that reflect biological complexity and includes a true positive reference dataset. In this study, we conducted a comprehensive evaluation of various quantitative proteomics designs, integrating different data analysis software tools and spectral library solutions.

## Experimental Procedures

### K562 Proteome Preparation for MS Analysis

K562 cells were grown in RPMI 1640 medium supplemented with 1% (v/v) L-glutamine and 10% fetal bovine serum (Sigma-Aldrich). After several passages, the cells were harvested by pelleting cells at 800g for 5 min at 4 °C. Cells were washed with cold PBS and gently resuspended before being centrifuged again. The supernatant was aspirated and washed again with PBS. After removal of the supernatant, the pellets were snap frozen in liquid nitrogen and stored at −80 °C.

A cell pellet equivalent to approximately 40 million cells was resuspended in 600 μl LiP buffer (100 mM Hepes pH 7.5, 150 mM KCl, and 1 mM MgCl2) and 0.5x cOmplete, EDTA-free protease inhibitor (Roche) and passed through a 27 G needle 10 times using a 1 ml syringe. The samples were incubated for 20 min on ice and then cleared by centrifugation (16,000*g* at 4 °C) for 4 min. Supernatant was retained in a new Eppendorf tube and the pellet was resuspended in 300 μl of LiP buffer for repeated lysis under the aforementioned conditions, including incubation and centrifugation. After centrifugation, supernatants were combined and protein amount was determined using a Pierce bicinchoninic acid (BCA) Protein Assay Kit according to manufacturer’s instructions.

### Limited Proteolysis Treatment with Staurosporine

K562 cell lysates were aliquoted in equivalent volumes in triplicate containing 120 μg sample and incubated for 15 min at 25 °C with staurosporine or vehicle control (1% dimethyl sulfoxide) at a final volume of 50 μl. An 8-point dose-response was used with the following staurosporine concentrations: 0, 0.1, 1, 10, 100, 1000, 10,000, and 50,000 nM. Proteinase K from *Tritirachium album* (Sigma-Aldrich) was added simultaneously to all the samples with the aid of a multichannel pipette, at a proteinase K: substrate mass ratio of 1:100, and incubated at 25 °C for 5 min. Digestion reactions were stopped by heating samples for 5 min at 98 °C in a thermocycler followed by the addition of sodium deoxycholate (DOC) (Sigma-Aldrich) to a final concentration of 5%. Samples were removed from heat and reduced for 30 min at 56 °C with 5 mM tris(2-carboxyethyl)phosphine hydrochloride followed by a 30 min incubation at room temperature (RT) in the dark with 15 mM iodoacetamide. Afterward, the iodoacetamide was quenched with the addition of 15 mM DTT. Samples were then diluted with 0.1 M ammonium bicarbonate to a final DOC concentration of 2.5% and digested for 2 h at 37 °C with lysyl endopeptidase (1:100 enzyme: substrate ratio; Wako). Samples were further diluted and digested for 16 h at 37 °C with trypsin (1:100 enzyme: substrate ratio) to a final DOC concentration of 1%. DOC was precipitated by the addition of formic acid (FA) to a final concentration of approximately 2% and centrifuged at 16,000g for 10 min. After transferring the supernatant to a new Eppendorf tube, the centrifugation was repeated to remove residual DOC and the digested samples desalted using Sep-Pak C18 cartridges (50 mg, 1 cc, Waters), using standard techniques with peptides being eluted with 35% acetonitrile (ACN) in 0.1% FA. Samples at this stage were split and dried under vacuum with 20 μg used for DIA measurements while 100 μg was used for TMT, assuming no loss during sample preparation. Two of the three replicates were used for TMT labeling. For high field asymmetric waveform ion mobility spectrometry (FAIMS) DIA testing only K562 cell lysates that were treated with vehicle control (1% dimethyl sulfoxide), and then subjected to the LiP-MS protocol were used.

For TMT labeling, anhydrous ACN was used to dissolve 0.5 mg of TMTpro 16 Plex reagent, which was then separated to three aliquots and dried under vacuum. Peptide samples for TMT were redissolved in 32 μl of 100 mM Hepes buffer (pH 8.5) in 30% ACN and vortexed. The samples were then transferred to tubes containing aliquoted and dried TMT (approximately 165 μg) and left to react for 1 h at 25 °C. A small amount of each sample was used to check the TMT labeling efficiency, while the rest of the sample was frozen at −20 °C (unquenched).

Labeling efficiency was checked on a pooled and C18 cleaned sample (50 mg, 1 cc, Waters) via 1D-LC MS. Afterward, the rest of the samples were quenched with 5 μl of 5% hydroxylamine for 15 min at RT, which was then acidified by the addition of 10 μl of 10% FA. All samples were pooled and dried down prior to resuspension in 1% ACN and 0.1% FA and cleaned using Sep-Pak C18 cartridges (200 mg, 3 cc, Waters) using standard techniques and the peptides eluted off with 50% ACN, in 0.1% FA.

### Offline High pH Fractionation

For TMT, approximately 800 μg was fractionated over an ACQUITY PRM PST CSH C18 column (1.7 μm 2.1 × 150 mm, Waters) using a gradient of 4 to 42% B, using eluents A (20 mM ammonium hydroxide, pH 10) and B (ACN) at a flow rate of 300 μl/min taking fractions every 30 s. Briefly, 96 fractions were subsequently concatenated to 24 samples (*e.g.*, fractions 1, 25, 49, and 73). For the creation of a spectral library for DIA, approximately 400 μg of pooled sample was fractionated to 84 fractions with a gradient of 2 to 38% ACN over 36 min, taking fractions every 30 s. The samples were concatenated to 12 fractions and dried under vacuum and stored at −20 °C until further measurement.

### LC-MS

Samples were analyzed using an EASY-nLC 1200 or Vanquish Neo UHPLC coupled to an Orbitrap Exploris 480 (Thermo Fisher Scientific). After reconstitution in 3% ACN and 0.1% FA, an amount corresponding to 2000 ng was injected (assuming no loss in the entire workflow). Peptides were separated on a 75 μm × 25 cm analytical column (packed in-house with 1.9 μm C18 resin; ReproSil-Pur 120 C18-AQ, Dr Maisch) applying a flow of 250 nl/min over a 120 min nonlinear gradient from 0 to 38% solvent B (0.1% FA in 90% ACN) and solvent A (0.1% FA, 3% ACN).

For DIA measurements, MS1 measurements were performed at 120,000 resolution (at 200 m/z) between 350 and 1650 m/z using an automatic gain control (AGC) target value of 3e6 charges and maximum injection time (maxIT) of 20 ms. Peptides were fragmented with a normalized higher energy collisional dissociation (HCD) of 27% using an Orbitrap resolution of 30,000, an AGC target value of 3000% and a maxIT of 54 ms. Forty variable windows were used to span the mass range from 350 to 1650 (m/z) using a 0.5 Da overlap with the first mass fixed to 250 (m/z) (supplemental Table S14).

Spectral library generation for DIA files were run with the same gradient though with an Orbitrap resolution of 60,000 and 15,000 for MS1 and MS2, respectively. A cycle time of 2s was used between subsequent MS1 acquisitions with the maxIT set to auto. Charges from 2 to 6 were fragmented with a normalized HCD energy of 27%. Isolation width of 4 (m/z) and dynamic exclusion of 14 s was implemented, as with DIA the first mass was fixed to 250 (m/z).

For FAIMS-DIA, samples were measured from −30 to −75 compensation voltage and without FAIMS in triplicate. All FAIMS measurements were operated under default settings, i.e., standard FAIMS resolution, total carrier gas flow was set to 4.6 (L/min), and the inner and outer electrode temperature set to 100 °C. The same LC-MS settings were otherwise identical as described above.

For TMT measurements a nonlinear gradient from 0 to 42% B was utilized due the more hydrophobic nature of TMTpro-tagged peptides. For TMT measurements, MS1 orbitrap resolution was 60,000 from a mass range of 400 to 1400. MS2 resolution was set to 45,000 with a maxIT of 100 ms and a normalized HCD collision energy of 32%. The first mass was fixed at 110 (m/z), and a normalized AGC target of 200% was used. Isolation window width was set to 0.7 Da to limit the number of peptides that would be coisolated during fragmentation. For TMT measurements without FAIMS, Advanced Peak Determination was turned off to minimize the acquisition of MS/MS spectra with coisolated precursors. Data-dependent scans were only performed on a single charge state per precursor. The cycle time was set to 3 s while the dynamic exclusion was set to 60 s.

For FAIMS-TMT, three compensation voltages were used, i.e., −35, −50, and −65, with a total cycle time of 3s. Two FAIMS methods were used, at high and low resolution corresponding to 45,000 and 15,000 resolutions, respectively. At high resolution, the same settings were used as described previously. For both FAIMS methods the dynamic exclusion list was shared between compensation voltages to avoid acquiring the same precursor. For FAIMS at low resolution, phase-constrained spectral deconvolution or Turbo-TMT FAIMS was utilized. Here, the maxIT was set to 22 ms, and the dynamic exclusion was decreased to 10 s. User defined lock mass (445.12003) was utilized for all non-FAIMS runs.

### MS Data Analysis

For DIA, data samples were analyzed with either Spectronaut 18 (version 18.5.231110.55695, Biognosys AG) or FragPipe (version 21.1) using MSFragger version 4.0 for spectra deconvolution and DIA-NN version 1.8.2 for feature quantification. Data were searched in three different ways: the DIA runs were directly searched against a proteome here coined “Direct DIA” (1). DIA runs which were supplemented with an additional custom-built spectral library generated from high pH fractions measured with DDA, were coined “Hybrid DIA” (2). “Classic DIA” whereby the DIA runs that are measured are used for quantification only and peaks are matched against a custom-built spectral library created as in “2” (3).

For both Spectronaut and MSFragger the digestion enzyme specificity was set to Trypsin/P and semispecific with up to two missed cleavages allowed. Search criteria included carbamidomethylation of cysteine as a fixed modification, as well as oxidation of methionine and acetylation (protein N terminus) as variable modifications. The data were searched against a FASTA file containing the canonical and reviewed human proteome (proteome ID: UP000005640) downloaded on 20201015 with proteinase K (*Engyodontium album*) and lysyl endopeptidase (*Lysobacter enzymogenes*) appended to the database (total of 20,372 protein entries). Peptide length was set to a minimum of seven. The false discovery rate (FDR) both at the peptide precursor and protein level were set to 1%).

For Spectronaut, default settings were used unless otherwise stated. Briefly mass calibration was set to dynamic with the ideal tolerance determined after a first pass calibration ( ± 40 ppm). Cross-run normalization was performed using local normalization, and the quantification window was not synchronized. Modified peptide was set as the minor peptide group for quantification. Quantification was performed on the MS2 level with imputation turned off. The libraries were generated using the library generation functionality of SpectroMine (version 4.2.230428.52329) using the Pulsar search engine platform with default settings. Briefly, fragment ions with an amino acid length <3 were removed, while a minimum of three and a maximum of six fragment ions/peptide were used. For samples analyzed in FragPipe, the workflow “DIA_SpecLib_Quant” was used with default settings unless mentioned otherwise. For the generation of a “Hybrid-DIA” and “Classic-DIA” datasets DDA files were searched using the DDA + data type, or wide windows, enabling the identification of additional coisolated peptides in the fragmentation window. RT calibration option was set to “automatic”, unless the library was generated from fractionated DDA data only, i.e., “Classic DIA”, in which the “ciRT” option was implemented. The precursor and fragment mass tolerance were both set to 20 ppm, though parameter optimization in MSFragger was also selected.

The following DIA-NN parameters were applied using the FragPipe workflow: --qvalue 0.01 --matrix-qvalue 0.01 --matrices --no-prot-inf --smart-profiling --no-quant-files --peak-center --no-ifs-removal --predictor --dl-no-rt --dl-no-im --strip-unknown-mods --report-lib-info –cfg. DIA-NN output pr_matrix files were used for downstream data analysis. Precursors were filtered to make sure that at least 50% of the samples had valid values. Modified peptides with more than one charge state were merged and only the most intense charge state (by sum intensity) was used. Spectronaut data were exported as peptide pivot tables, and MS2 intensity values between 0 and 1.5 were considered as missing values and replaced with “NaN”. Peptide tables from both Spectronaut and FragPipe were loaded to Perseus (2.0.3.1) (18) and data imputation was performed using default settings (downshifted by 1.8 and with a width of 0.3) and applied to each column separately. After imputation a standard two-tailed *t* test, assuming unequal variance was performed. Only peptides above a fold change log2 > 0.46 and *p*-value <0.01 were used for fitting to a sigmoidal curve across the entire dose response range, as described in Piazza *et al*. 2020 (5).

TMT data were searched using either Thermo Proteome Discoverer (3.0.1.27) and Sequest HT or with FragPipe (v21.1) using MSFragger version 4.0. With Proteome Discoverer, a precursor mass tolerance of 10 ppm and a fragment mass tolerance of 0.02 Da were set. Min and max peptide length were set to 7 and 30, respectively. Trypsin/P with semi-tryptic specificity with up to two missed cleavages was allowed. TMTpro to the peptide N terminus and lysine side chains was set as a static modification as was carbamidomethylation of cysteine residues. Oxidation on methionine residues was set as a variable modification. INFERYS rescoring node was implement, while percolator was used for determining FDRs. The data were searched against the same database as described above. Reporter ion abundance was based on intensity. Only peptides with a coisolation threshold of less than 50 and a S/N threshold above 10 were used. Normalization was performed on the total peptide amount. Peptides and proteins were filtered after analysis to only include peptides and proteins of high confidence (1% FDR). For analysis of TMT data with FragPipe and MSFragger, IonQuant (1.10.12) was used for quantification. Median centering normalization was applied as the normalization strategy. Semi-tryptic searches and modifications were used as above but with a peptide length from 7 to 50. Otherwise, default settings were used using the TMT-16 plex workflow in FragPipe, with mass calibration and parameter optimization implemented using an initial precursor mass tolerance of ± 20 ppm. Only peptides with 100% channel occupancy were used, as such no imputation was necessary. A standard two-tailed *t* test, assuming unequal variance was performed. Data wrangling was performed as above to generate Spectronaut-like report and candidate files that could be used for fitting to a sigmoidal curve across the entire dose response range, as described in Piazza *et al*. 2020 (5).

For receiver operating characteristic (ROC) curves visualization, sensitivity and specificity were calculated as followed: sensitivity corresponds to the true positive rate and measure how many true positives are identified.

Sensitivity is calculated as follows:

Sensitivity = True Positives (TP)/(False Negatives (FN) + True Positives (TP)).

In this context, True Positives (TP) are the kinases correctly identified, and False Negatives (FN) are the kinases incorrectly identified as non-kinases.

Specificity instead measures the number or True Negatives that are correctly identified and is calculated as follows:

Specificity = True Negatives (TN)/(True Negatives (TN) + False Positives (FP))

Here, True Negatives (TN) are non-kinases correctly identified as such, and False Positives (FP) are non-kinases incorrectly identified as kinases.

The variable on the X-axis of ROC curves is calculated as 1–Specificity.

## Results

### Experimental Design for Assessing LiP-MS Efficiency in Detecting Protein Structural Changes

**Fig. 1:**
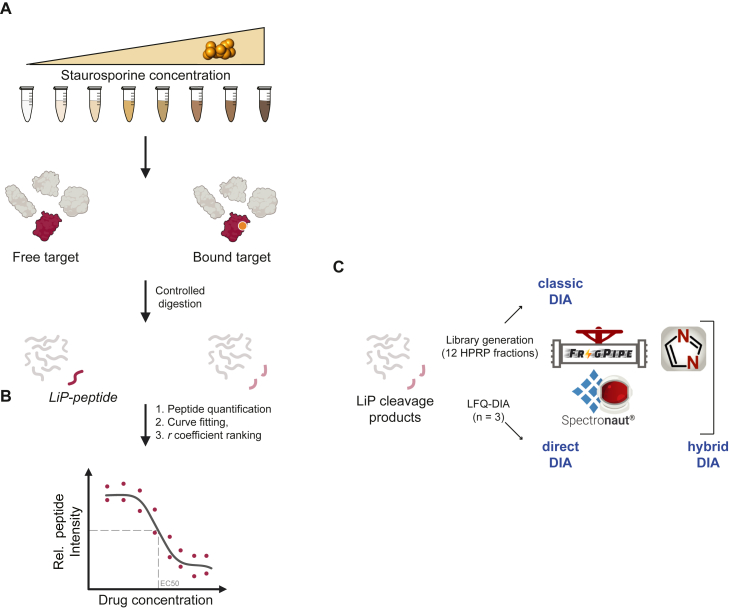
**Design of the LiP-MS benchmarking experiment and data analysis workflows**. *A*, principle and experimental design of the LiP-MS protein–drug interaction detection experiment. It includes a dose-response analysis approach using the drug staurosporine as a test case. Sample preparation for MS analysis follows a controlled proteolysis reaction with broad specific protease to produce LiP-peptides with abundance proportional to staurosporine dose. *B*, after mass spectrometry data extraction and quantification, we calculated dose-response curves correlating staurosporine drug concentration and peptide intensities and then rank them by their correlation coefficient *r*. *C*, three DIA data acquisition modes (direct-DIA, hybrid-DIA, and classic-DIA) are used for processing with the software tools Spectronaut and FragPipe (with DIA-NN in library free mode). For direct-DIA peptide cleavage products measured for each of the eight staurosporine concentrations in triplicates are measured by DIA-MS and directly processed with label-free quantification (LFQ) with both Spectronaut and FragPipe. In classic-DIA mode DDA-MS data from the same samples are acquired from prefractionated 12 high pH reverse phase (HPRP) fractions to build project specific libraries generated with the database search engines MSFragger-DIA and Pulsar. DIA-MS runs were then searched and quantified by LFQ with both Spectronaut and FragPipe. The hybrid-DIA option combines classic-DIA and direct-DIA. LiP-MS, limited proteolysis coupled with mass spectrometry; DIA, data-independent acquisition; DDA, data-dependent acquisition.

To create a benchmark experiment for systematically assessing protein structural changes using LiP-MS, we focused on detecting protein–drug interactions. We prepared native protein lysates from cultured human cells and introduced the drug staurosporine into this proteome in a dose-response manner (Fig. 1*A*). Staurosporine was chosen as it is a well-characterized *pan-*kinase inhibitor (5, 19, 20), known to bind nearly all 522 human ATP-binding kinases with minimal off-target effects. With these known staurosporine targets serving as a true positive reference dataset, we evaluated both the number of peptides and proteins quantified by each DIA analysis workflow and their accuracy in matching expected staurosporine targets.

Each native lysate sample, spiked with a defined dose of staurosporine underwent limited proteolysis, followed by quantitative proteomics to measure the resulting structure-specific cleavage peptides. LiP-peptide hits were identified within the complex peptide background by selecting signals that exhibited a staurosporine dose-response curve fitting a log-logistic function across the entire concentration range (Fig. 1*B*) (5).

### Comparative Evaluation of DIA Data Analysis Workflows for LiP-MS

We experimented with three distinct DIA data analysis workflows, each incorporating different classes of spectral libraries. First, we generated a project-specific library from DDA data, acquired through offline peptide prefractionation. This integrates prior knowledge of peptide fragmentation patterns and retention times, restricting data searches to a predefined precursor ion list. We called this approach classic DIA, as it is widely supported by most DIA analysis tools and is a proven strategy for managing DIA data complexity.

In contrast, we also employed a direct DIA search approach, where deconvoluted DIA spectra were searched against a proteome database without relying on preexisting libraries. This method, referred to as direct DIA search, allows for a broader and more unbiased analysis. Additionally, we explored a hybrid DIA mode, which combines the advantages of a project-specific DDA library with direct DIA searches. Our project-specific DDA library consisted of twelve peptide fractions separated by high pH reverse-phase chromatography (Fig. 1*C*).

We evaluated the performance of each spectral library design using a combination of the most common DIA software suites capable of processing classic, direct, and hybrid spectral libraries. Specifically, we utilized the open-source FragPipe platform, which integrates MSFragger-DIA (10) for peptide identification and DIA-NN for quantification (DIA-NN library free) (13). Additionally, we tested the commercial software Spectronaut (Fig. 1*C*).

To benchmark these tools, we compared Spectronaut against the open-source FragPipe, focusing on the combination of two software solutions (FragPipe and Spectronaut) with three spectral library types. This resulted in six distinct DIA data analysis workflows, providing a comprehensive evaluation of different approaches.

### Performance on Proteome and Peptide Identification

**Fig. 2:**
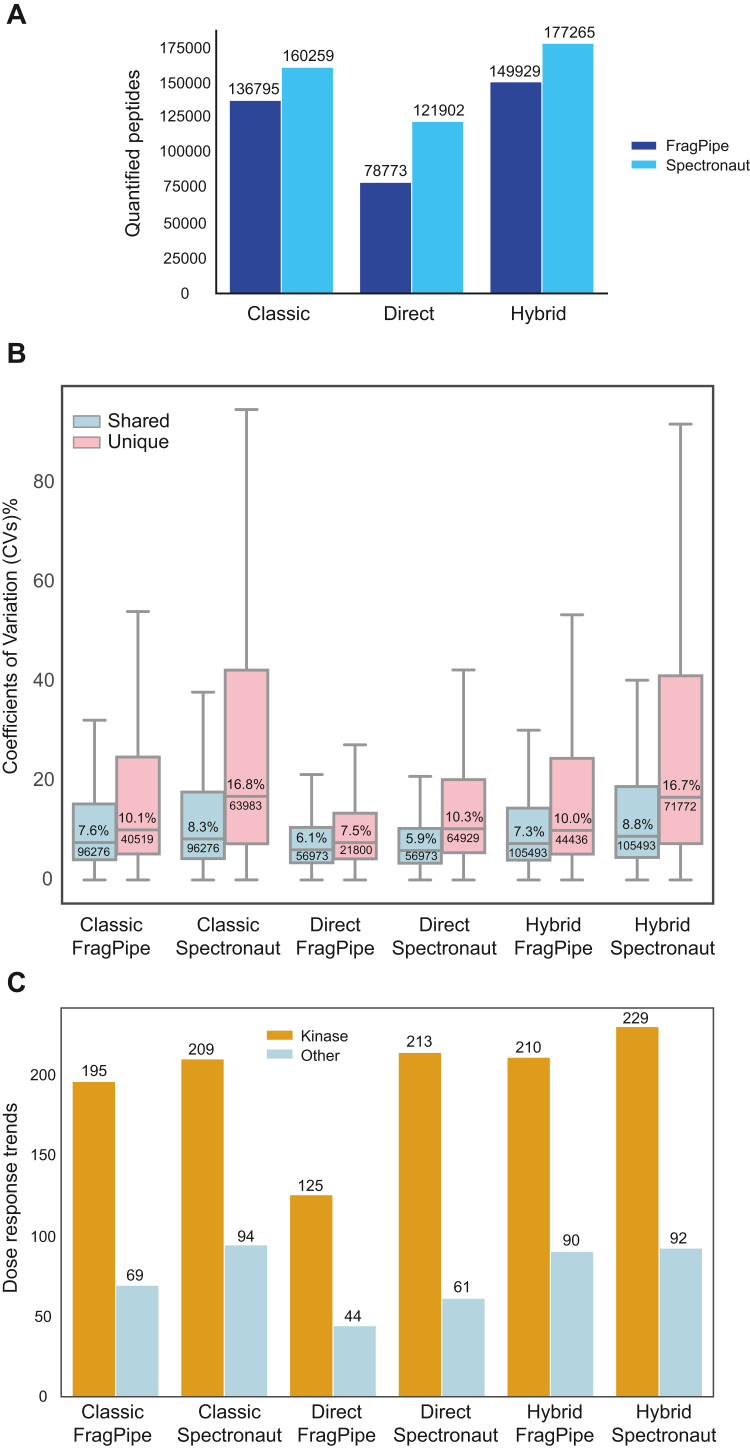
**LiP-MS peptide quantification quality with multiple DIA-MS modes**. *A*, bar plots of the quantified peptides with the different DIA quantification modes and software. *B*, box plots of peptide coefficients of variation (% CV). *Blue boxes* show overlapping peptides shared between the FragPipe and Spectronaut analysis tools. *Pink box* plots illustrate unique peptides quantified exclusively by each specific tool. The box in each box plot captures the interquartile range with the top and bottom edges representing Q1 and Q3, respectively. The median is the horizontal line within the box. The whiskers lengths extend to the minima or maxima within 1.5 times the interquartile range below Q1 or above Q3. The median value (in % CV) and the number of peptides of the group are reported in inside the box plot. *C*, bar plots of fitted sigmoidal trends of LiP peptides with r > 0.75 of protein kinases (Kinase) or proteins of other classes (Other). LiP-MS, limited proteolysis coupled with mass spectrometry; DIA, data-independent acquisition; CV, coefficient of variation.

Using Spectronaut, we quantified 32% more peptides (Fig. 2*A*) and 25% more protein groups (supplemental Fig. S1*A*) when extracting peptide ion features against a spectral library built from twelve high pH fractions (classic DIA) compared to using direct DIA. The hybrid DIA approach identified 17,006 additional peptides and 303 more proteins than classic DIA, offering an ideal balance between experimental specificity, completeness, and increased proteome depth.

Spectronaut consistently demonstrated the highest sensitivity, quantifying more peptides than FragPipe across all three spectral library types (Figs. 2*A* and supplemental Fig. S1*A*). However, despite Spectronaut’s broader coverage, FragPipe exhibited higher quantification precision, as indicated by lower coefficient of variation (CVs) across all spectral library options. The lowest CVs were observed with direct DIA processing using both Spectronaut (median CV 10.3%) and FragPipe (median CV 7.5%). Peptides shared between FragPipe and Spectronaut generally had low CVs (6.1% for FragPipe and 5.9% for Spectronaut), suggesting that these shared features are of high quality and accuracy (Fig. 2*B*).

Overall, this analysis shows that both FragPipe and Spectronaut provide good precision and sensitivity, with the highest proteome coverage achieved using Spectronaut with a hybrid library, while FragPipe with direct DIA offers the best quantitative precision (supplemental Tables S1–S6).

### Drug Dose-Response Fitting of LiP Peptides

We next investigated how quantification precision and analytical depth impact the correct quantification of biological data, a critical benchmark criterion. To do this, we calculated staurosporine dose-response curves for each peptide signal quantified using our six different DIA data analysis workflows and calculated the correlation coefficient (*r*) to a sigmoidal trend in the experimental profiles. The number of peptides with an r higher than 0.75, indicating reliable staurosporine dose-response curves, ranged from 321 peptides (Spectronaut with hybrid DIA) to 169 peptides (FragPipe with direct DIA) (supplemental Tables S1–S6).

Five of the six data analysis approaches detected over 195 staurosporine LiP-peptides mapping to more than 66 protein kinases, with minimal differences between Spectronaut and FragPipe (Fig. 2*C*, supplemental Fig. S1*B*, and supplemental Table S1). The combination of direct DIA with FragPipe detected only 125 LiP peptides corresponding to 53 protein kinases (Fig. 2*C* and supplemental Fig. S1*B*), likely due to MS-Fragger-DIA quantifying 78,773 of peptides compared to the 121,902 quantified by Spectronaut with direct DIA. These results highlight that using additional DDA runs for spectral library building significantly enhances drug targeting sensitivity with FragPipe, while Spectronaut’s direct DIA produced the most staurosporine-responsive peptide sigmoids among kinases, with limited additional benefit from an experimentally derived library. The correlation coefficient (*r*) remained the best parameter to discriminate drug protein target in our data, when we tested alternative metrics for the same scope (supplemental Fig. S1*C*).

### Benchmarking FragPipe and Spectronaut for LiP-MS Based Drug-Target Identification

**Fig. 3:**
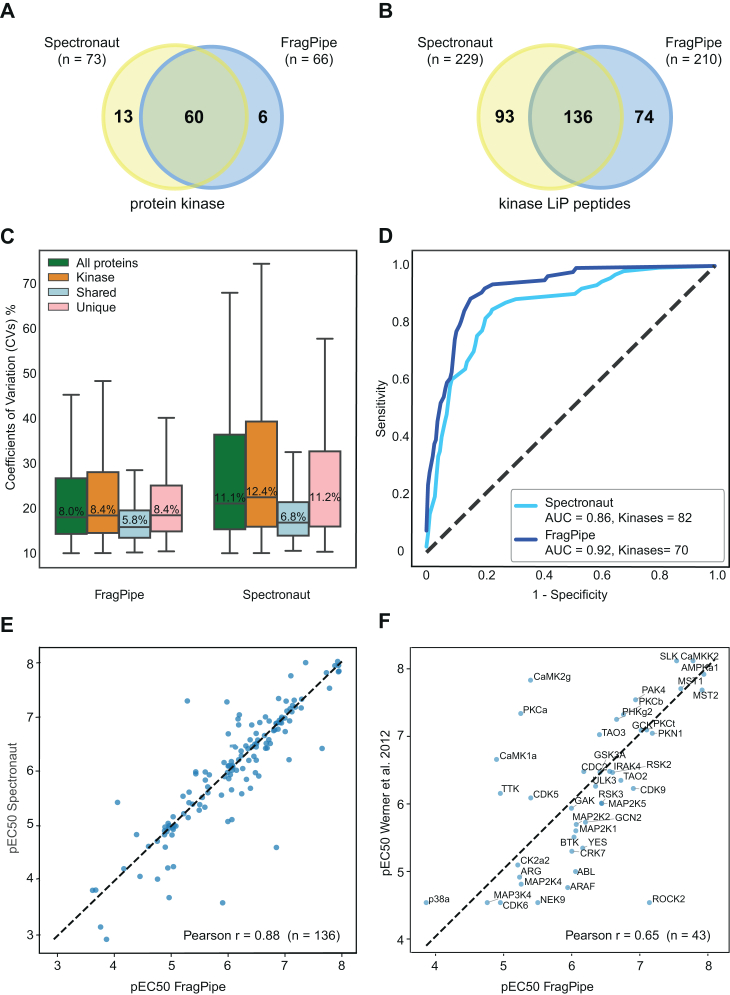
**Comparing FragPipe and Spectronaut with the LiP-MS hybrid-DIA quantification mode**. *A*, total number of kinase protein targets and common protein kinase targets of staurosporine found using Spectronaut or FragPipe. *B*, total number of kinase peptide targets and common peptide kinase targets of staurosporine found using Spectronaut or FragPipe. *C*, box plots of peptide coefficient of variations (% CVs) of different protein categories. Protein kinases (kinase) or proteins of other classes (all proteins) or kinase peptides shared between the FragPipe and Spectronaut analysis tools (shared) or unique kinase peptides quantified exclusively by each specific tool (unique). The *box* in each box plot captures the interquartile range with the top and bottom edges representing Q1 and Q3, respectively. The median is the *horizontal line* within the box. The whiskers length extends to the minima or maxima within 1.5 times the interquartile range below Q1 or above Q3. *D*, receiver operating characteristic (ROC) curves of staurosporine protein interactions, their respective area under the curve (AUC) values, and number of kinase identified (kinases) measured by DIA LiP-MS with a hybrid-DIA library and processed by Spectronaut (*cyan*) or FragPipe (*blue*). The *dashed line* represents a random classifier. The ground truth is represented by the 253 protein kinases quantified with the hybrid-DIA library approach. *E*, Pearson correlation (r) of the concentrations of drug at which we observed a 50% of the maximum LiP peptide intensities (visualized as −log_10_ effective concentration or pEC50) extrapolated from the dose-response curves of hybri−d-DIA LiP-MS data quantification with Spectronaut or FragPipe. *F*, Pearson correlation (r) of the concentrations of drug at which we observed a 50% of the maximum LiP peptide intensities (visualized as −log_10_ effective concentration or pEC50) from LiP-MS dose-response data (pEC50 Hybrid FragPipe) and pEC50s reported from Kinobeads data (Werner *et al*. 2012). DIA, data-independent acquisition; LiP-MS, limited proteolysis coupled with mass spectrometry.

To further assess the performance of Spectronaut and FragPipe-based DIA data analysis methods in identifying protein kinases binding staurosporine, we ranked the protein candidate targets using the correlation coefficient (r) score, considering the 522 annotated human protein kinases in KinHub (21) (http://www.kinhub.org) (supplemental Table S2) as true positives (supplemental Tables S1–S6). We focused on the hybrid DIA workflow, which produced the highest number of dose-response sigmoidal curves for both software suites (Fig. 2*C*). With both Spectronaut and FragPipe, known staurosporine targets ranked among the top candidate peptides and proteins (supplemental Fig. S1*D*). While Spectronaut identified more kinase targets (73) and more LiP peptides (229) compared to FragPipe (66 kinase targets and 210 LiP-peptides), both methods showed comparable performance (Fig. 3, *A* and *B*). FragPipe identifies fewer false positives than Spectronaut with 92 (Spectronaut) and 90 (FragPipe) LiP-peptides and 72 (Spectronaut) and 63 (FragPipe) proteins other than kinases (supplemental Table S1). Notably, a significant portion of kinases (60) and LiP-peptides (136) were detected by both Spectronaut and FragPipe (Fig. 3, *A* and *B*). The shared kinase targets exhibited lower median peptide CVs compared to unique hits, emphasizing the importance of high quantitative quality for accurate drug-target identification by LiP-MS (Fig. 3*C*).

The performance of LiP-MS for drug-target identification slightly improved with FragPipe, as the area under the receiver operating characteristic curve (AUC-ROC) increased from 0.86 to 0.92 (Fig. 3*D*). Additionally, LiP-MS drug dose-response titrations allowed us to estimate the half-maximal effective concentration (EC50) of compound binding sites in whole-cell lysates, which is the concentration of staurosporine where we observed 50% of the maximum LiP-peptide signal. By comparing the EC50 values derived from sigmoidal curves quantified with FragPipe and Spectronaut, we found a strong correlation, indicating consistent and reliable quantification across both software platforms (Fig. 3*E* and supplemental Table S7). Our EC50 values extrapolated from LiP peptides are also in good accordance with published data (Fig. 3*F*).

Overall, our benchmarking demonstrates that Spectronaut offers high sensitivity, while FragPipe excels in prioritizing LiP-MS hits for drug target discovery. Spectronaut's higher sensitivity generally comes at the cost of reducing selectivity, allowing slightly more false positive hits (Fig. 2*C*, supplemental Fig. S1*B*, and supplemental Table S1). Similar conclusions were drawn when analyzing the direct DIA workflow (supplemental Fig. S2, *A*–*E*).

### A Workflow Combining LiP-MS with TMT-Isotopic Labeling

#### Optimization of TMT-Based Quantification for LiP-MS

**Fig. 4:**
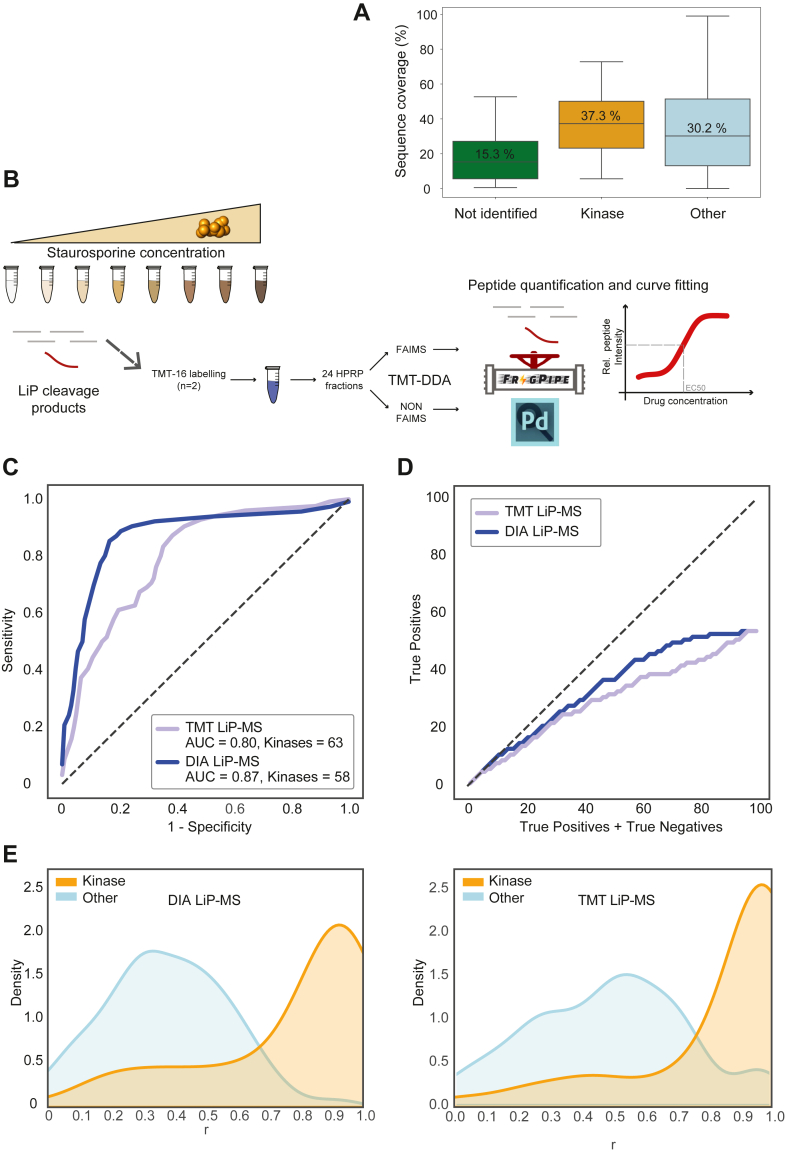
**Performance of TMT and label-free peptide quantification workflows for LiP-MS**. *A*, box plots of protein sequence coverage relative to the following protein groups (from *left* to *right*): protein kinases not detected in this experiment, protein kinases quantified in this experiment, and proteins with other functions than kinases. These data are relative to LiP-MS quantified in direct DIA mode using Spectronaut. *B*, experimental design and principle for data analysis of the TMT-LiP-MS workflow for drug-target deconvolution. *C*, receiver operating characteristic (ROC) curves of staurosporine protein interactions, their respective area under the curve (AUC) values, and number of kinases identified (Kinases) measured with DIA-MS, a direct library and FragPipe for data extraction (DIA LiP-MS) or measured with TMT-DDA (TMT LiP-MS). The *dashed line* represents a random classifier. The ground truth is represented by the 185 protein kinases detected by the two quantification methods used here. *D*, true positive rate evaluation for TMT LiP-MS and DIA LiP-MS on kinase target identification for staurosporine. True positive hits in the top 100 candidates are shown as a function of the number of true and false positives in the candidate list for TMT LiP-MS or DIA LiP-MS. The *dashed line* indicates a perfect candidate list consisting of only true positives (slope = 1), where true positives are protein kinases, as staurosporine is a promiscuous binder of protein kinases. *E*, distribution of LiP peptides for DIA LiP-MS (*left*) and TMT LiP-MS (*right*) over the r correlation coefficient for kinases (kinase) and non-kinase (other) peptides. The densities of the two populations are normalized by the area under the curve to account for different population sizes, facilitating comparison. The density plots compare kinase and other peptides from the direct DIA experiment analyzed with FragPipe and from the TMT experiment analyzed with Proteome Discoverer, illustrating their distribution based on the correlation coefficient (r). LiP-MS, limited proteolysis coupled with mass spectrometry; DIA, data-independent acquisition; DDA, data-dependent acquisition; TMT, tandem mass tag.

LiP-MS enables the identification and quantification of protein interactions at the peptide level, providing resolution down to the ligand-binding sites. However, this level of detail can also be a limitation, as protein-drug interactions may remain undetected due to insufficient protein sequence coverage. In our benchmarking staurosporine experiment using DIA-MS, we observed that successfully detected kinase targets exhibited higher median protein sequence coverage compared to the entire proteome, and particularly in contrast to undetected kinases (Fig. 4*A*). This observation aligns with previous findings (5), and underscores the importance of high sequence coverage for accurate drug-target identification by LiP-MS.

Based on this, we hypothesized that peptide undersampling, which leads to lower protein coverage, might reduce the identification of kinase targets. To address this, we tested TMT isobaric labeling as an alternative quantification strategy, aiming to enhance sensitivity (*e.g.*, proteomic depth) and improve proteome sequence coverage. We labeled the same peptide samples from the staurosporine benchmark experiment with TMT-16 reagent (Fig. 1) and adjusted the experimental design to include two 8-dose replicates of drug-treated lysates within the same TMT-plex. We then analyzed 24 offline fractionated peptide samples (Fig. 4*B*), using the same instrument time as for label-free quantification in direct DIA mode (Fig. 1*C*).

Before comparing DIA LiP-MS and TMT LiP-MS based quantification, we optimized TMT data acquisition parameters that are particularly relevant for acquiring TMT data at high resolution (22) and that have been previously used for drug-target identification (23). We tested FAIMS separation (24) with multiple compensation voltages to increase the number of precursors entering the mass spectrometer (see Experimental Procedures). While this approach reduced ratio compression, it also resulted in attenuated signal intensity, due to the extended ion flight path. Additionally, we tested the phase-constrained spectrum deconvolution enhanced resolution option (25) (also known as TurboTMT), which allows a high MS2 scan rate with reliable quantification of TMT reporter ions (26). Both FAIMS and TurboTMT are expected to improve protein and peptide coverage.

Indeed, LiP-MS analysis depth increased significantly with the use of TMT and offline fractionation (TMT LiP-MS), yielding a 65% increase in the number of peptides, a 43.4% increase in the number of proteins, and a 47% increase in the number of kinases compared to direct DIA (supplemental Tables S3 and S8). FAIMS combined with TMT-DDA quantification (FAIMS TMT LiP-MS, supplemental Table S9) further increased the number of quantified peptides by 15.6% and proteins by 4.4%. The TurboTMT option tripled the number of acquired spectra, resulting in a 45.4% increase in quantified peptide groups (FAIMS TurboTMT LiP-MS, supplemental Table S10), although the number of protein groups only modestly increased by 9.4% compared to standard high-resolution TMT without FAIMS (supplemental Fig. S3*A*). The medians of the CVs for peptide ions ranged from 2.4% to 4.8% (supplemental Fig. S3*B*), significantly lower than the CVs observed with DIA quantification with Spectronaut (10.3%) (Fig. 2*B*), reflecting the high precision of TMT isobaric labeling.

While the TurboTMT option provided the highest peptide quantification among the TMT methods, it also exhibited the lowest quantification precision (median CV 4.8%), although the differences between the three TMT options were subtle. The lowest CVs were obtained by processing the data without FAIMS and with TurboTMT turned off, resulting in a median CV below 3%. The features quantified across all TMT acquisition options generally had low CVs compared to uniquely quantified peptides, indicating high quantification quality (supplemental Fig. S3*B*). We also noted that while FAIMS and TurboTMT boosted the number of kinase LiP-peptides (supplemental Fig. S3*B*) and true positive kinases identified, they also allowed a relatively larger number of false positive hits (supplemental Table S1). In summary, both FAIMS and TurboTMT combined with TMT-DDA increased protein and peptide sensitivity but did not significantly improve data quality.

Due to the observed benefits of FAIMS in combination with TMT, we also evaluated the performance of LiP-MS with DIA, both with and without FAIMS. We found no significant increase in peptide identifications with the addition of FAIMS, regardless of the compensation voltage applied (supplemental Fig. S3*C*). While FAIMS has demonstrated advantages in conjunction with DIA for short gradients (27), our findings align with previous studies showing that FAIMS-DIA offers limited benefits for longer gradients, as observed here for LiP-MS samples (28).

Our results confirmed that TMT isobaric labeling significantly increases the number of peptides quantified in a LiP-MS experiment compared to DIA-MS. Quantification precision is also markedly improved, as evidenced by much lower CV values (reduced from approximately 10% to 3%) when using TMT. A slight benefit in identification numbers was observed with the use of FAIMS in TMT-based experiments.

### Comparison of LiP-MS Quantification by TMT and DIA Workflows with Fixed Instrument Time

We next examined whether the increased quantification precision obtained with TMT translates into improved identification of protein kinases binding staurosporine in our LiP-MS drug-target deconvolution benchmark experiment. We compared the performance of the LiP-MS predictor when using TMT-MS versus DIA-MS for mass spectrometry data acquisition, focusing on fitting sigmoidal trends with peptide intensity profiles. For this comparison, we selected the TMT-MS option without FAIMS and the DIA-MS workflow using a direct library with FragPipe analysis, as these configurations yielded the lowest CV values in their respective groups.

When ranking the drug-response sigmoidal curves by their correlation coefficients, we found that the AUC-ROC curve for TMT (0.80) was lower than that for DIA (0.87) (Fig. 4*C*), despite the higher precision of peptide quantification with TMT (supplemental Fig. S3*B*). Further investigation revealed that LiP-MS coupled with DIA-MS more effectively discriminates true positive staurosporine targets among top-ranking candidate proteins than TMT-MS (Fig. 4*D*). Additionally, we analyzed the distribution densities of correlation coefficients (r) for LiP peptides mapping to kinase and non-kinase peptides, comparing TMT and DIA-MS quantification. While LiP-kinase peptides consistently exhibited higher r-coefficients than non-kinase peptides in both scenarios, these values were notably higher and the peaks sharper in TMT analysis, indicating greater precision. However, this precision advantage was offset by the fact that non-kinase peptides also displayed higher r-values, reducing the effectiveness of TMT in improving kinase identification as reflected in the ROC curve (Fig. 4*E*). When quantifying LiP-MS data with TMT, the use of FAIMS or FAIMS-TurboTMT resulted in a smaller AUC of the ROC curve, indicating a decrease in predictive performance of drug targets (supplemental Fig. S3*D*). Similar results were obtained when analyzing TMT data with freeware software solutions offered by FragPipe instead of Proteome Discoverer (supplemental Fig. S4*A* and supplemental Tables S12 and S13).

The kinase features identified using TMT and DIA are generally overlapping with the two quantification strategies with some specificities. For instance, 22 unique protein kinases with TMT LiP-MS and 15 unique protein kinases with DIA LiP-MS (supplemental Fig. S4*B*), corresponding to 142 and 87 LiP-peptides, respectively (supplemental Fig. S4*C*). When we compared the EC50 values extrapolated from LiP peptides quantified by DIA-MS or TMT-MS with an orthogonal approach, we discovered that the EC50s tend to be in mild-to-good accordance with similar estimates made with chemoproteomics methods based on chemical probes (29) (Kinobeads). Since Kinobeads are specifically designed to quantitatively assess binding affinities, the good correlation between binding parameters estimated by LiP-MS and those measured with Kinobeads (Pearson r = 0.73 for DIA-MS - supplemental Fig. S4*D* and Pearson r = 0.62 for TMT-MS - supplemental Fig. S4*E*), demonstrated that both TMT LiP-MS and DIA LiP-MS provide reliable estimate of the relative strength of interactions between drugs and their protein targets.

In summary, we performed a LiP-MS benchmark assay using staurosporine, a drug with well-characterized protein targets, and an experimental design with equivalent instrument time to compare the quantitative performances of DIA and TMT-based workflows. Our findings demonstrate that while TMT coupled with LiP-MS provides more sensitive target identification with excellent precision, the DIA workflow is preferable for its good accuracy and generally lower costs.

## Discussion

In this study, we performed a comprehensive benchmarking of quantitative proteomics workflows for LiP-MS, focusing on detecting protein structural changes and drug–protein interactions. A key observation from our analysis is that DIA and TMT isobaric labeling in combination with LiP-MS, are similarly effective at identifying drug targets in our benchmark dataset, as recently observed for thermal proteome profiling experiments (30). TMT labeling facilitated the quantification of more peptides, proteins, and kinases, and resulted in significantly lower CVs, underscoring its ability to generate high-quality quantitative data. However, the increased sensitivity of TMT did not always correspond to improved accuracy in identifying true drug targets. For instance, in the case of staurosporine-binding kinases, DIA-MS was more effective in identifying true positive targets, as reflected by a higher AUC-ROC and stronger correlation of peptide intensity profiles to drug dose-response curves. This discrepancy between sensitivity and accuracy can be explained by the inherent features of each approach. TMT quantification benefits from reduced missing values and improved precision, yet it is vulnerable to ratio compression and coisolation artifacts, which can affect target identification in complex proteomes. On the other hand, DIA-MS, although generally offering lower peptide coverage, leads to greater accuracy in detecting protein–drug interactions.

For DIA-based LiP-MS, the use of an experiment-specific spectral library remains advantageous for maximizing sensitivity. Interestingly, our analysis showed that freely available software solutions, such as FragPipe, offer a competitive alternative to commercial options like Spectronaut. Ultimately, the choice between FragPipe and Spectronaut presents a trade-off: FragPipe provides maximum precision, while Spectronaut offers maximum sensitivity, particularly in direct searches that do not rely on experiment-specific spectral libraries. FragPipe-based analyses contains fewer false positives resulting in a better performance in drug-target deconvolution using ROC curves.

In conclusion, this study underscores the importance of selecting the appropriate LiP-MS quantification strategy based on the specific experimental goals. By benchmarking these workflows using a well-characterized drug–protein interaction dataset, we provide actionable guidelines for the proteomics community, especially for those involved in drug discovery and structural biology. Future advancements in mass spectrometry instrumentation and data analysis algorithms, such as the Astral mass spectrometer or advanced ion mobility separations, are expected to enhance the detection of low-abundance proteins and, further increase protein sequence coverage, potentially reducing the need for TMT labeling (31). The Astral-MS, in particular, is anticipated to improve sensitivity by identifying more peptides and increasing sequence coverage, expanding the application of LiP-MS across diverse biological contexts.

## Experimental Design and Statistical Rationale

An 8-point dose-response staurosporine treatment (with staurosporine concentrations of 0, 0.1, 1, 10, 100, 1000, 10,000, and 50,000 nM) was performed in triplicate on K562 cell lysates resulting in a total of 24 samples. For TMT due to the limit in number of channels available for labeling, only two of the three replicates could be used for the TMT experiments, with labeling performed using a TMTpro 16 Plex reagent kit. TMT labels within the TMTplex as well as the DIA acquisition run order was randomized to reduce potential batch effects.

## Data Availability

The mass spectrometry proteomics data have been deposited to the ProteomeXchange Consortium via the PRIDE (32) partner repository with the dataset identifier PXD055927.

## Supplemental data

This article contains supplemental data.

## Conflict of interest

The authors declare no competing interests.

## Supplementary data

S1_Koudelka_Classic_MSF

S2_Koudelka_Classic_SN

S3_Koudelka_Hybrid_MSF

S4_Koudelka_Hybrid_SN

S5_Koudelka_Direct_MSF

S6_Koudelka_Direct_SN

S7_Koudelka_Top_Ranking

S8_Koudelka_TMT_LiP_PD

S9_Koudelka_TMT_FAIMS_LiP_PD

S10_Koudelka_TURBO_TMT_LIP_PD

S11_Koudelka_TMT_LiP_FragPipe

S12_Koudelka_TMT_FAIMS_LiP_FragPipe

S13_Koudelka_TURBO_TMT_LiP_FragPipe

S14_Koudelka_DIA_Windows

Koudelka_Supplementary_table1_ERROR MODEL

Koudelka_Supplementary_table2_KINASE LIST

Koudelka_Supplementary_table3_IDS

Sx_Koudelka_Column_Descriptions4_SuppDataTables1_13

Koudelka_Revised_F1S LiP-MS protein quantification quality with multiple DIA-MS modes. *A*, bar plots of the quantified proteins with the different DIA quantification modes and software. *B*, bar plots of proteins belonging to LiP peptides displaying sigmoidal trends with r of the fitting > 0.75 . *Blue bars* refer to protein kinases, orange bars to proteins of other classes. *C*, comparison of correlation coefficient (*r*), effective concentration EC50 and peptide FDR as metrics for discriminating staurosporine targets. Each figure shows the distribution of LIP-MS hits mapped to kinase (Kinase) and non-kinase (Other) proteins. Data was analyzed using Direct DIA and FragPipe. *D*, true positive rate evaluation for all six DIA LiP-MS modes tested on kinase target identification for staurosporine. True positive hits in the top 100 candidates are shown as a function of the number of true and false positives in the candidate list. The *solid black line* indicates a perfect candidate list consisting of only true positives (slope = 1), where true positives are protein kinases, as staurosporine is a promiscuous binder of protein kinases

Koudelka_Revised_F2S Comparing FragPipe and Spectronaut with the LiP-MS direct-DIA quantification mode *A*, total number of kinase protein targets and common protein kinase targets of staurosporine found using Spectronaut or FragPipe in direct-DIA mode. *B*, total number of kinase peptide targets and common peptide kinase targets of staurosporine found using Spectronaut or FragPipe in direct-DIA mode. *C*, receiver operator characteristic (ROC) curves of staurosporine protein interactions, their respective area under the curve (AUC) values, and number of kinases identified (Kinases) measured by DIA LiP-MS with a direct-DIA library and processed by Spectronaut or FragPipe. The dashed line represents a random classifier. The ground truth is represented by the 185 protein kinases detected with the direct-DIA library approach. *D*, Pearson correlation (r) of the concentrations of drug at which we observed a 50% of the maximum LiP peptide intensities (visualized as - log10 effective concentration - pEC50) extrapolated from the dose-response curves of direct-DIA LiP-MS data quantification with Spectronaut or FragPipe. *E*, Pearson correlation (r) of the concentrations of drug at which we observed a 50% variation of the maximum LiP peptide intensities (visualized as - log10 effective concentration - pEC50) from LiP-MS dose-response data (pEC50 Direct FragPipe) and pEC50s reported from Kinobeads data (Werner *et. al.* 2012).

Koudelka_Revised_F3S Optimization of TMT-based quantification for LiP-MS. *A*, quantified peptides with the assessed TMT LiP-MS quantification variants: TMT (TMT LiP-MS), TMT with FAIMS (FAIMS-TMT LiP-MS) and TMT with FAIMS and TurboTMT® (FAIMS-TurboTMT® LiP-MS). All options increased both peptide and protein identifications compared to DIA. *B*, box plots of peptide coefficients of variations (CV). Cyan boxes show overlapping peptides shared among the three different TMT data acquisition settings. Pink box plots illustrate unique peptides quantified exclusively by one specific TMT data acquisition method. The box in each box plot captures the interquartile range with the top and bottom edges representing Q1 and Q3 respectively. The median is the horizontal line within the box. The whiskers length extends to the minima or maxima within 1.5 times the interquartile range below Q1 or above Q3. *C*, number of peptides and proteins quantified when combining LiP-MS with FAIMS-DIA with different negative compensation voltages. Values correspond to DMSO treated samples (vehicle control) measured with three biological replicates. *D*, receiver operator characteristic (ROC) curves of staurosporine protein interactions, their respective area under the curve (AUC) values, and number of kinases identified (Kinases) measured with the three assessed TMT LiP-MS quantification variants. The dashed line represents a random classifier. The ground truth is represented by the 185 protein kinases detected by the two quantification methods used here.

Koudelka_Revised_F4S Quantitative analysis of drug targets captured by TMT-LiP-MS. *A*, receiver operator characteristic (ROC) curves of staurosporine protein interactions, their respective area under the curve (AUC) values, and number of kinases identified (Kinases) measured with TMT LiP-MS data analysed with the commercial software Proteome Discoverer or the freeware software FragPipe. The dashed line represents a random classifier. *B*, total number of kinase protein targets and common protein kinase targets of staurosporine found by DIA LiP-MS or TMT LiP-MS. *C*, total number of kinase peptide targets and common peptide kinase targets of staurosporine found by DIA LiP-MS or TMT LiP-MS. *D*, Pearson correlation (r) of the concentrations of drug at which we observed a 50% of the maximum LiP peptide intensities (visualized as - log10 effective concentration - pEC50) from LiP-MS dose-response data (pEC50 DIA LiP-MS) and pEC50s reported from Kinobeads data (Werner *et. al.* 2012). *E*, Pearson correlation (r) of the concentrations of drug at which we observed a 50% variation of the maximum LiP peptide intensities (visualized as - log10 effective concentration - pEC50) from LiP-MS dose-response data (pEC50 TMT LiP-MS) and pEC50s reported from Kinobeads data (Werner *et. al.* 2012).

F1_Publication License
