# Supplementary material for: Benchmarking of Quantitative Proteomics Workflows for Limited Proteolysis Mass Spectrometry
Source: Mol Cell Proteomics. 2025 Mar 13;24(4):100945. doi: 10.1016/j.mcpro.2025.100945 (PMC12022698; doi:10.1016/j.mcpro.2025.100945)
Supplement: S7_Koudelka_Top_Ranking [file mmc7.pdf]

Classic_FragPipe

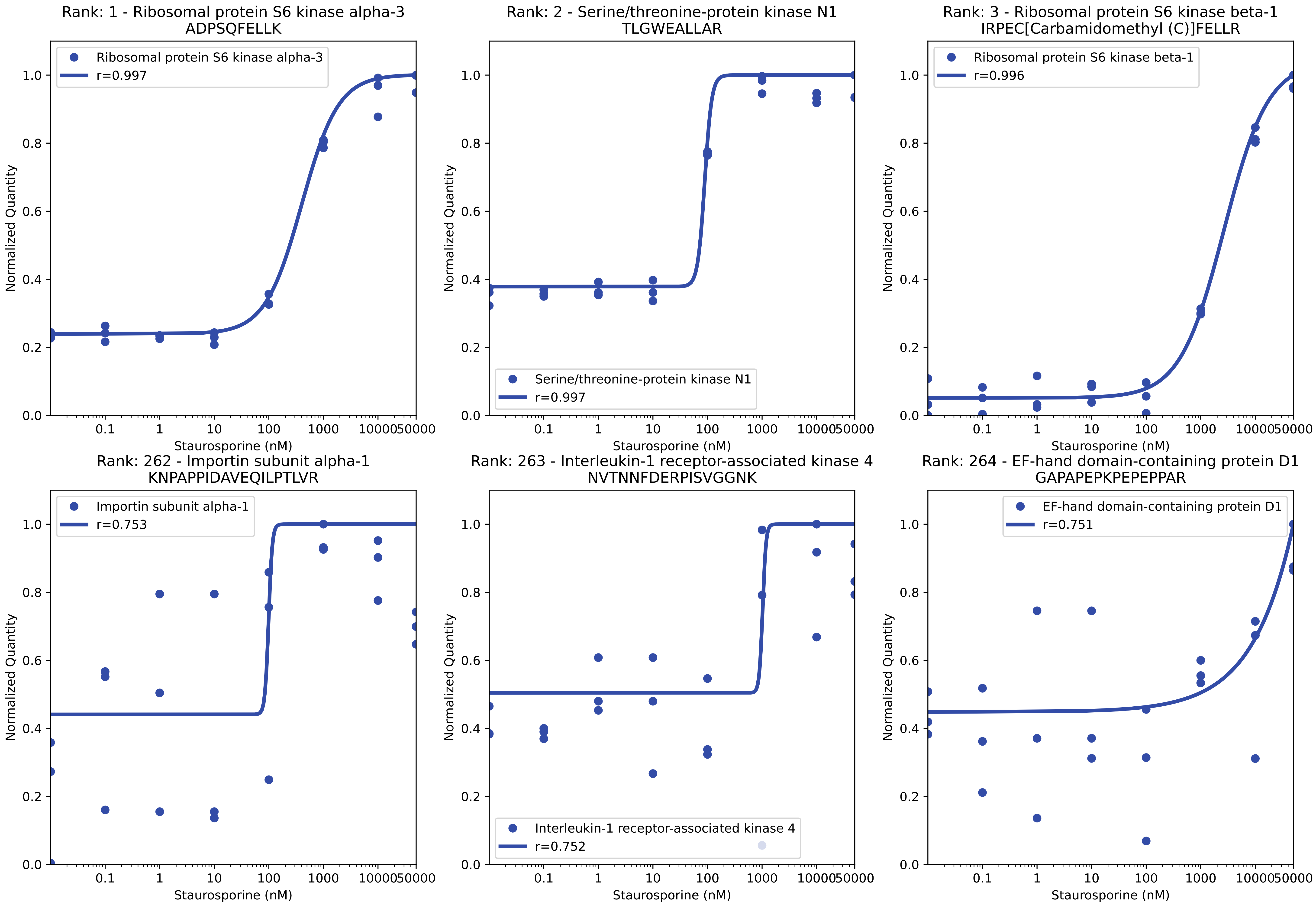

Classic_Spectronaut

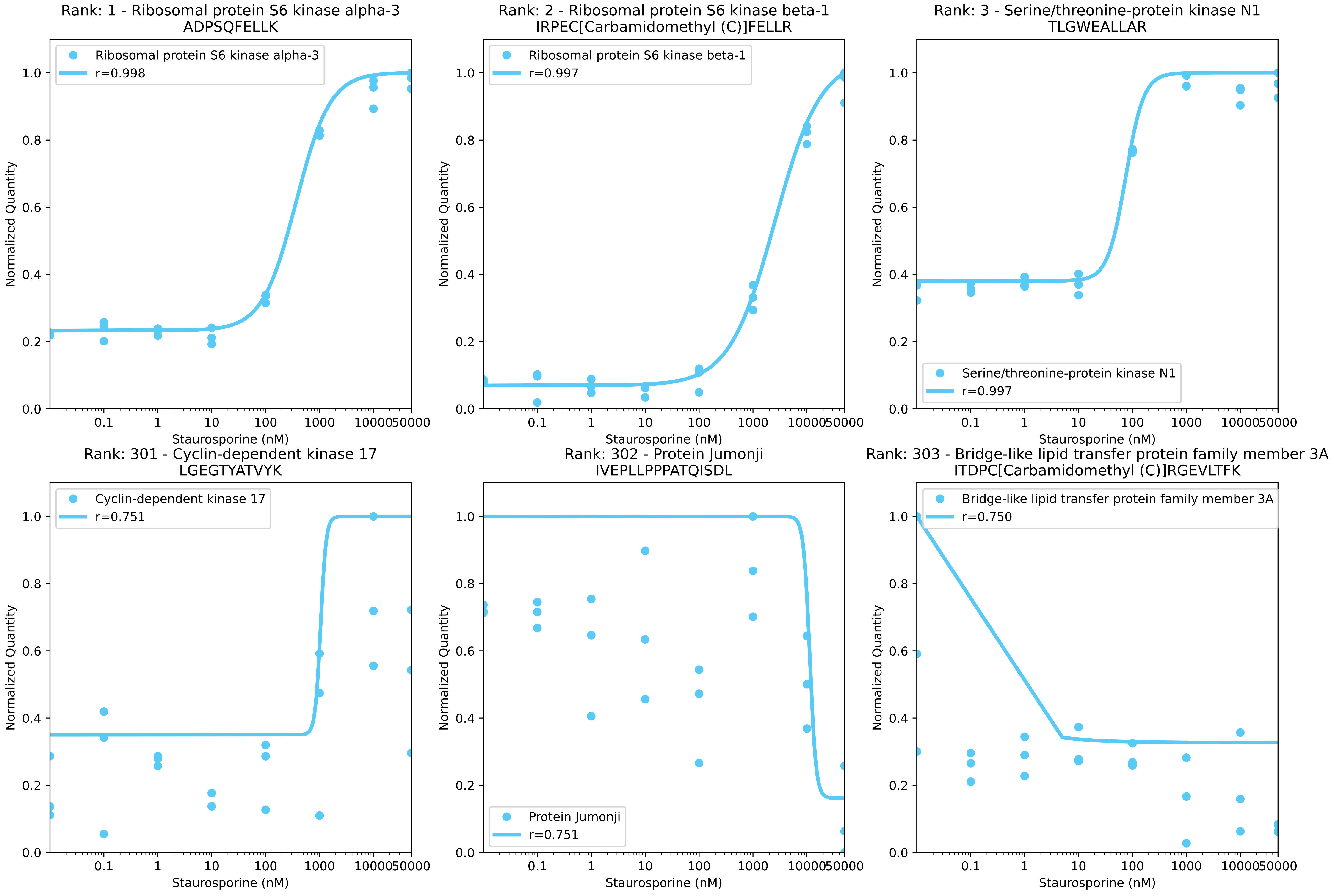

Direct_FragPipe

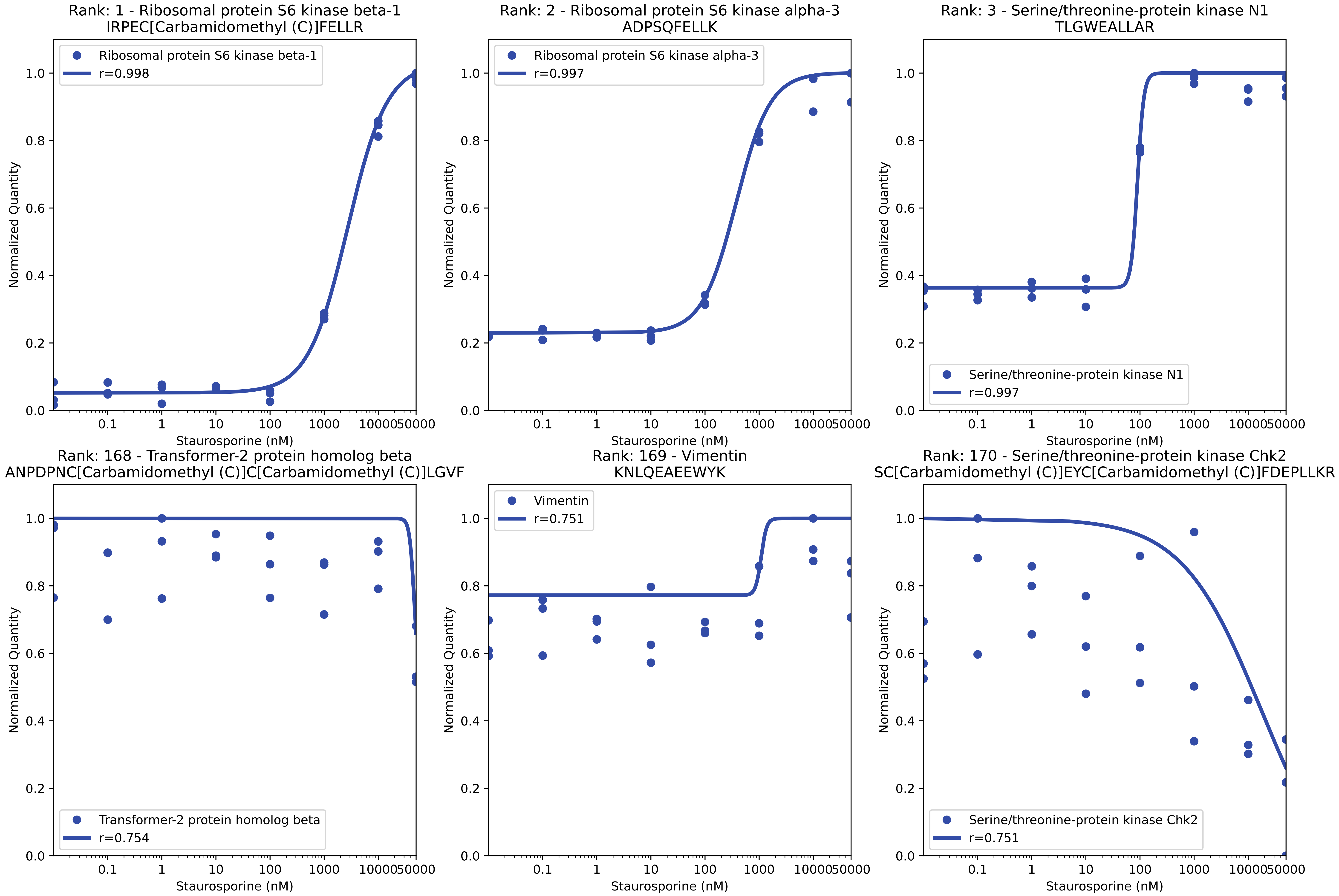

Direct_Spectronaut

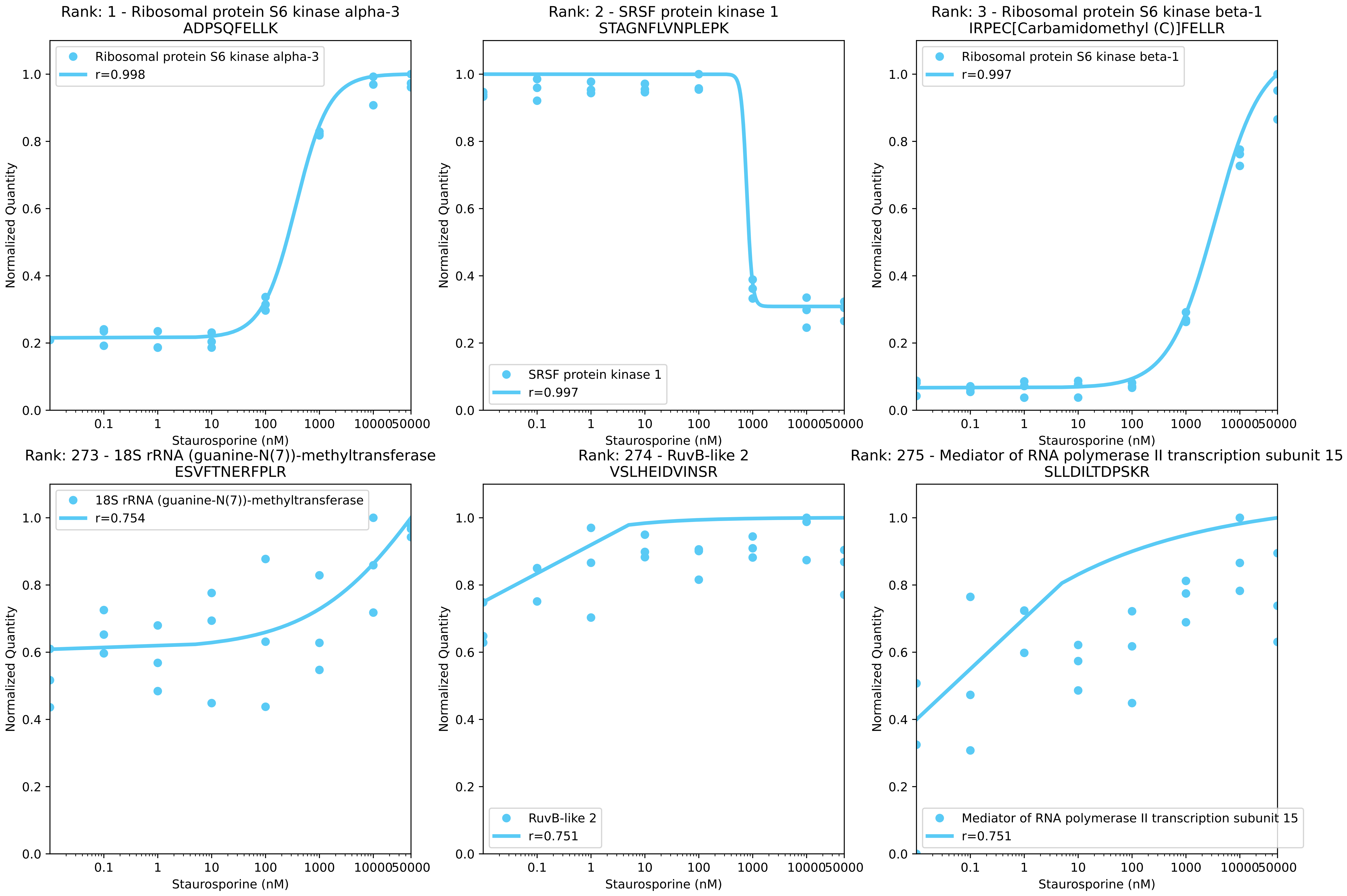

Hybrid_FragPipe

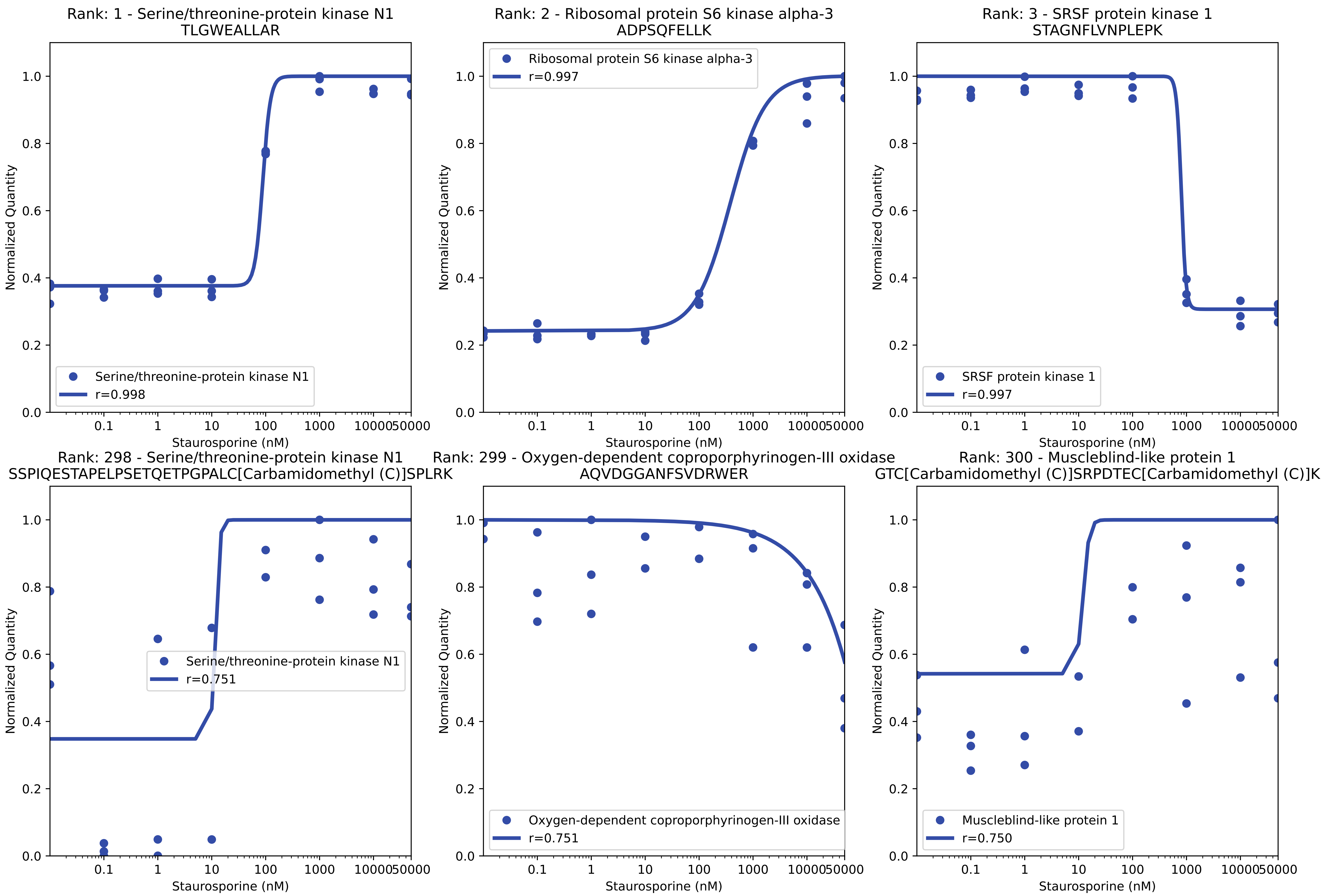

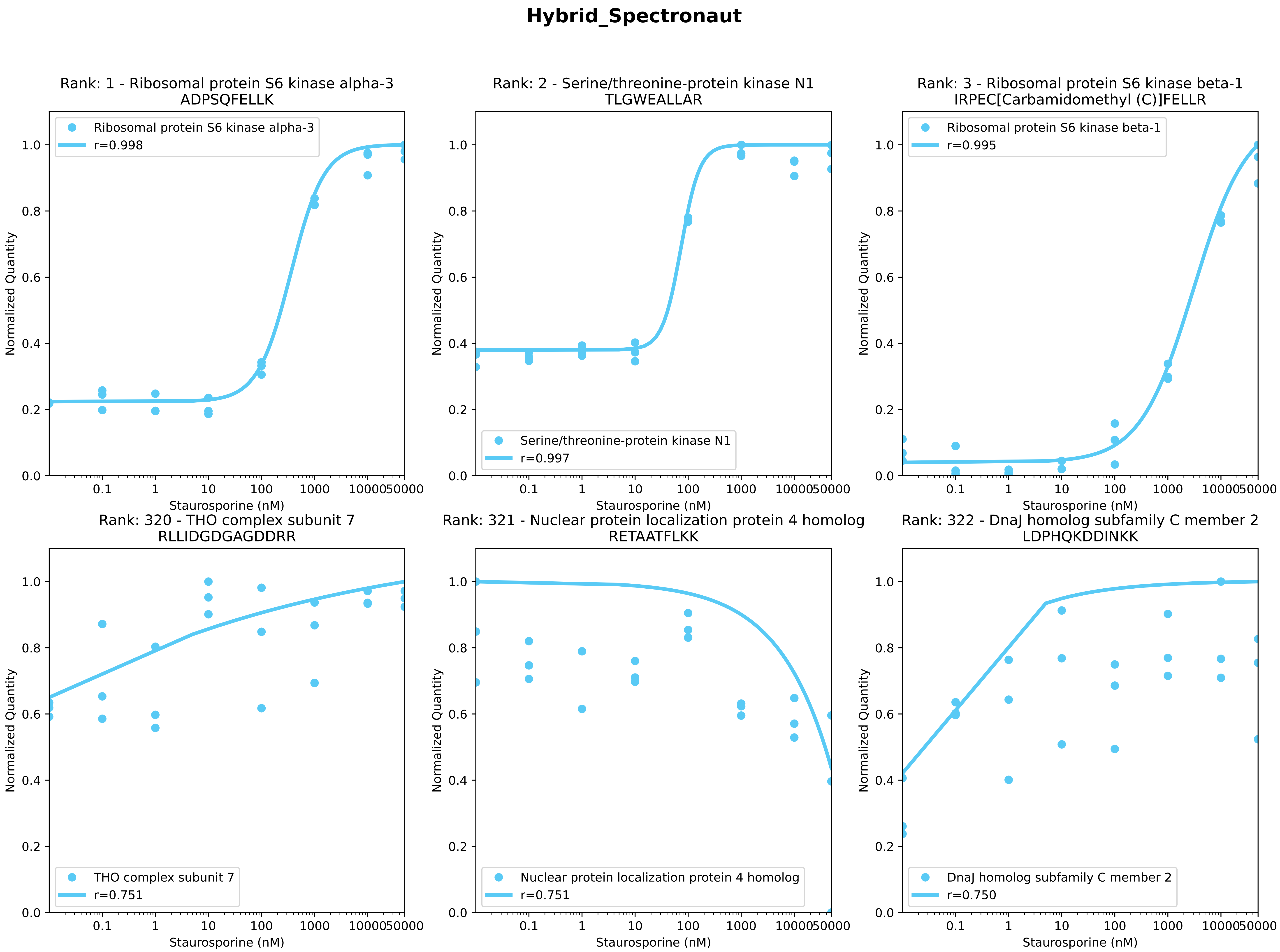
Hybrid_Spectronaut
Rank: 1 - Ribosomal protein S6 kinase alpha-3
ADPSQFELLK
Ribosomal protein S6 kinase alpha-3
r=0.998
Rank: 2 - Serine/threonine-protein kinase N1
TLGWEALLAR
Serine/threonine-protein kinase N1
r=0.997
Rank: 3 - Ribosomal protein S6 kinase beta-1
IRPEC[Carbamidomethyl (C)]FELLR
Ribosomal protein S6 kinase beta-1
r=0.995
Rank: 320 - THO complex subunit 7
RLLIDGDGAGDDRR
THO complex subunit 7
r=0.751
Rank: 321 - Nuclear protein localization protein 4 homolog
RETAATFLKK
Nuclear protein localization protein 4 homolog
r=0.751
Rank: 322 - DnaJ homolog subfamily C member 2
LDPHQKDDINKK
DnaJ homolog subfamily C member 2
r=0.750
Normalized Quantity
Staurosporine (nM)
0.0
0.2
0.4
0.6
0.8
1.0
0.1
1
10
100
1000
10000
50000
