# Supplementary figures and images for: Benchmarking of Quantitative Proteomics Workflows for Limited Proteolysis Mass Spectrometry
Source: Mol Cell Proteomics. 2025 Mar 13;24(4):100945. doi: 10.1016/j.mcpro.2025.100945 (PMC12022698; doi:10.1016/j.mcpro.2025.100945)

**A**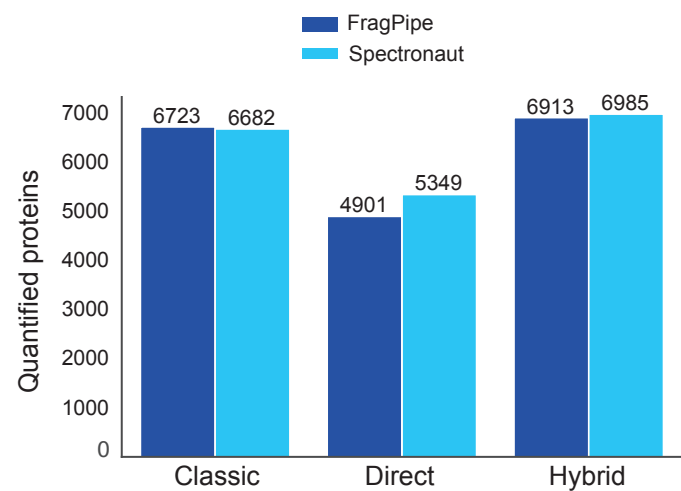**B**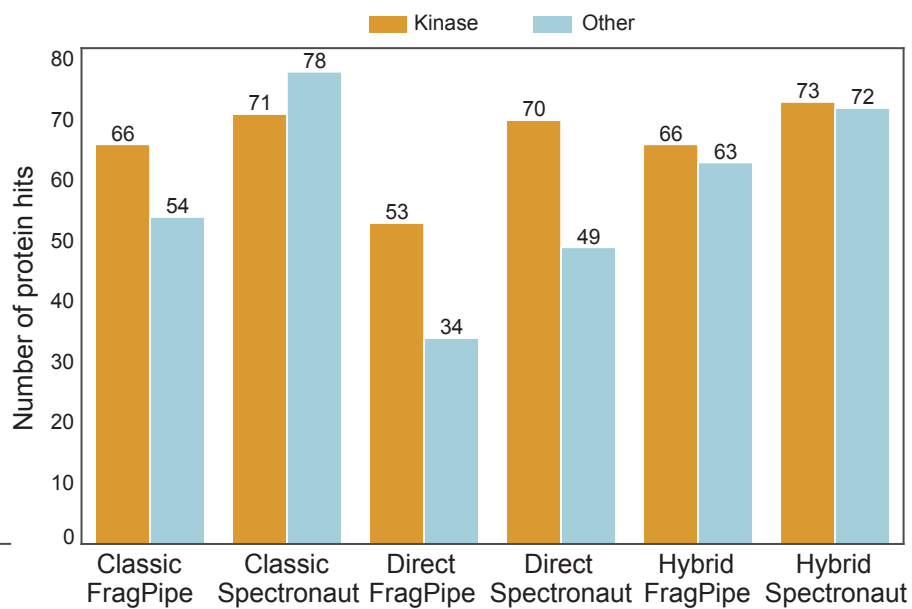**C**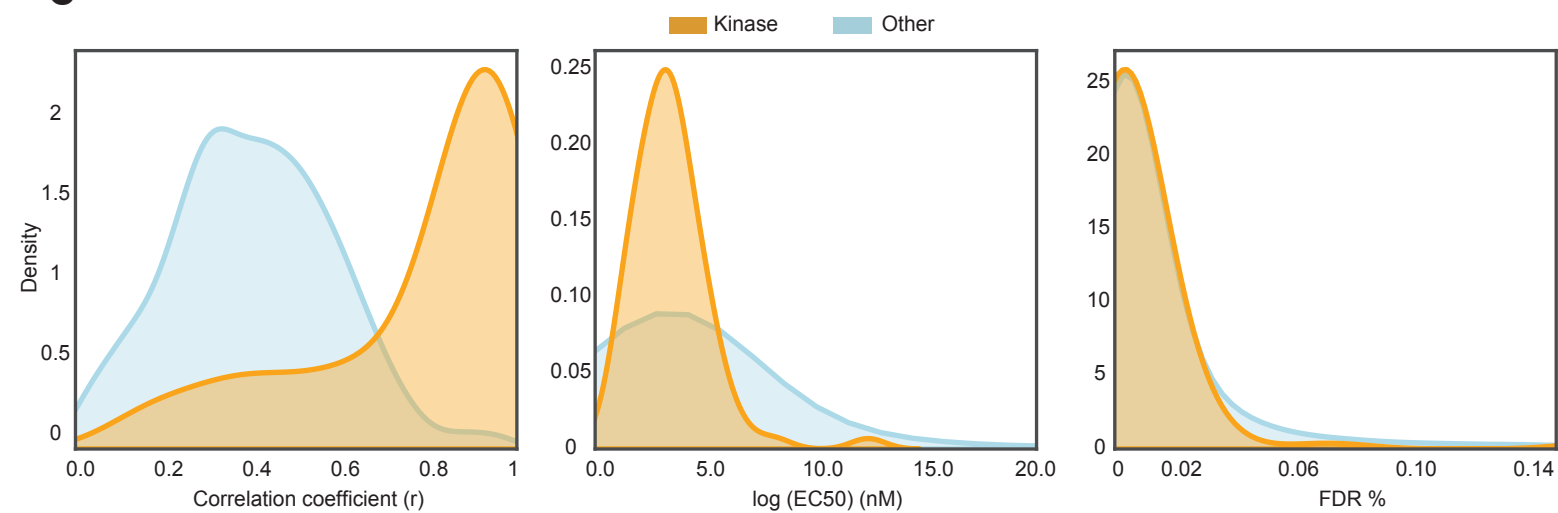**D**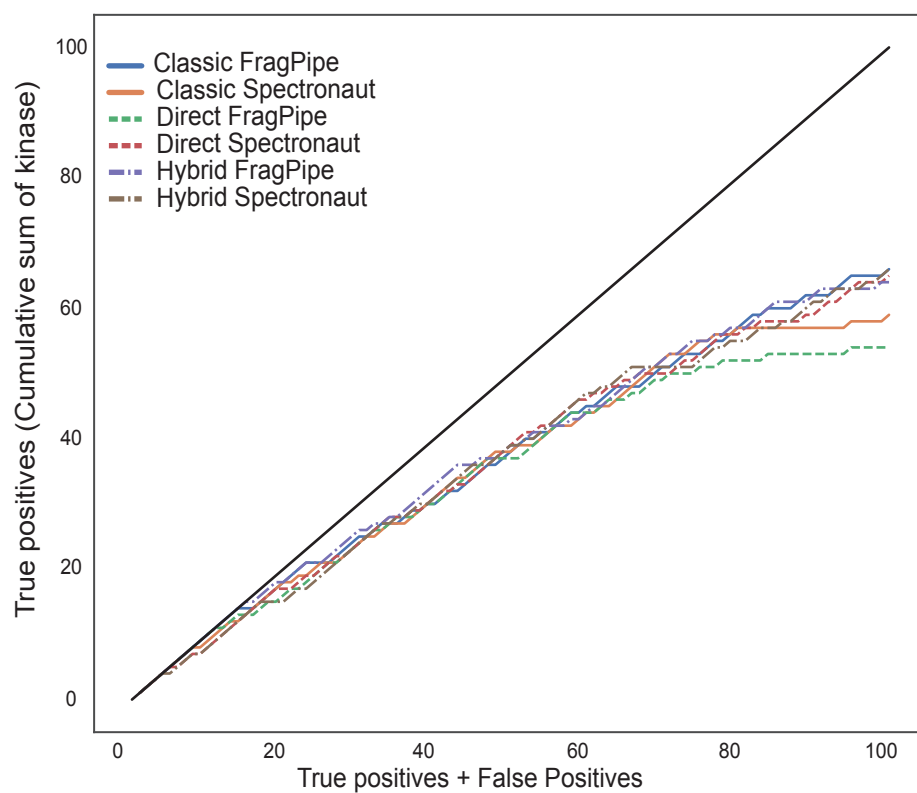

Figure S1

Supplement: Koudelka_Revised_F1S — LiP-MS protein quantification quality with multiple DIA-MS modes. A, bar plots of the quantified proteins with the different DIA quantification modes and software. B, bar plots of proteins belonging to LiP peptides displaying sigmoidal trends with r of the fitting > 0.75 . Blue bars refer to protein kinases, orange bars to proteins of other classes. C, comparison of correlation coefficient (r), effective concentration EC50 and peptide FDR as metrics for discriminating staurosporine targets. Each figure shows the distribution of LIP-MS hits mapped to kinase (Kinase) and non-kinase (Other) proteins. Data was analyzed using Direct DIA and FragPipe. D, true positive rate evaluation for all six DIA LiP-MS modes tested on kinase target identification for staurosporine. True positive hits in the top 100 candidates are shown as a function of the number of true and false positives in the candidate list. The solid black line indicates a perfect candidate list consisting of only true positives (slope = 1), where true positives are protein kinases, as staurosporine is a promiscuous binder of protein kinases [file mmc19.pdf]

**A**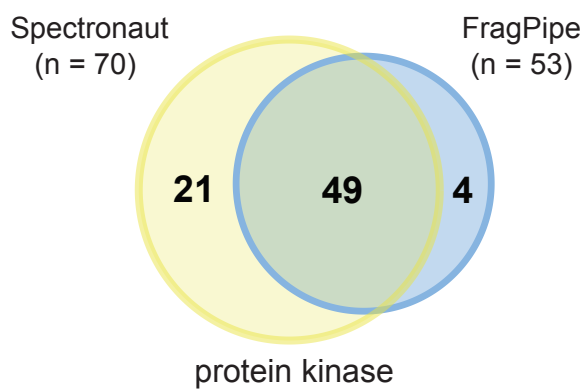**B**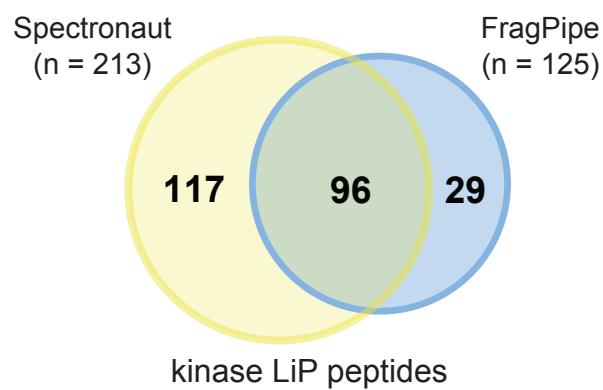**C**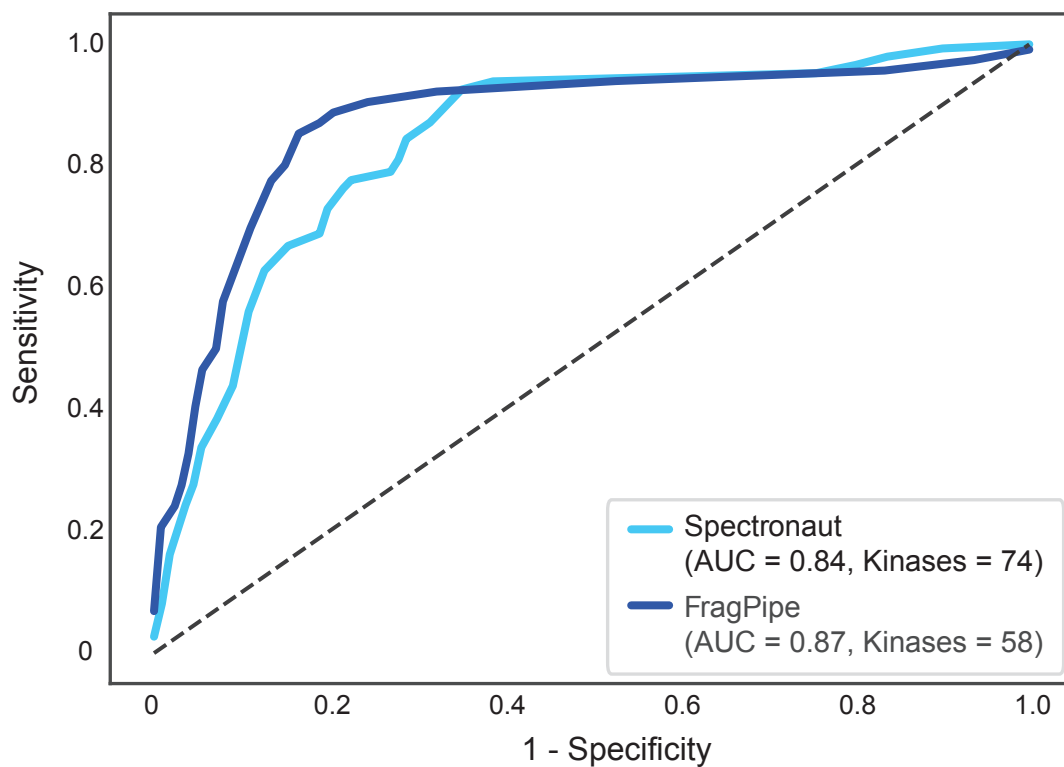**D**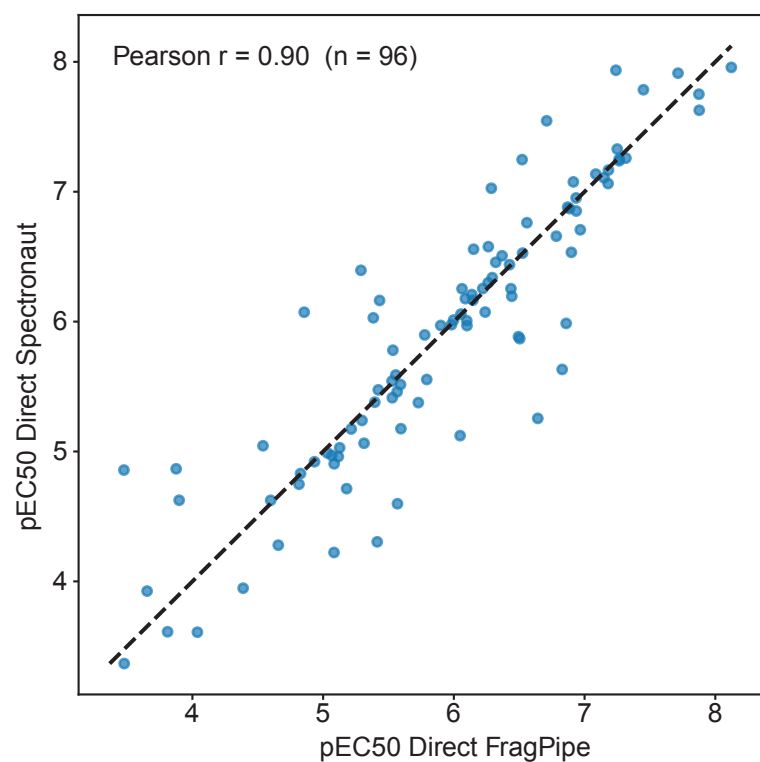**E**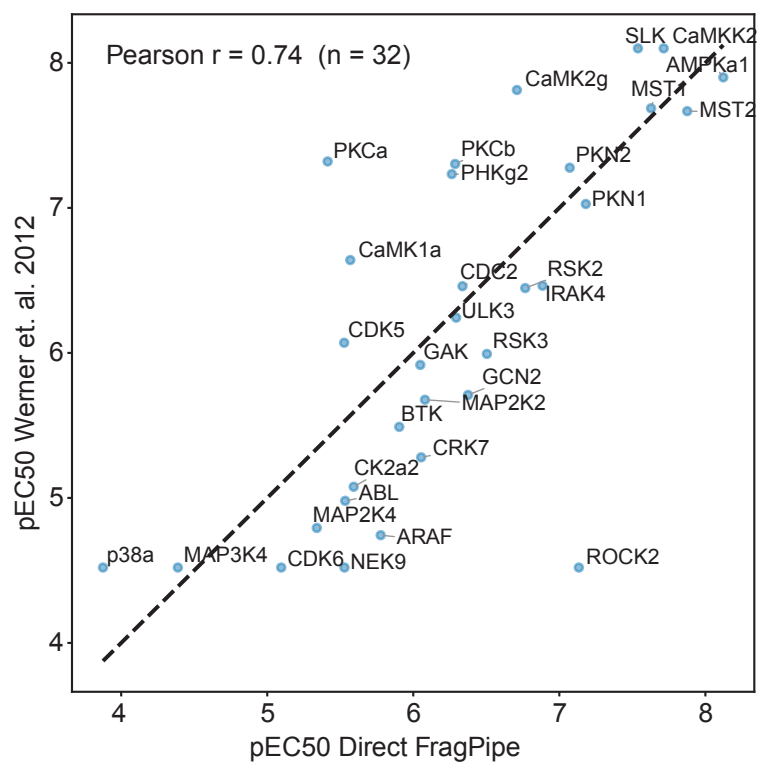

Figure S2

Supplement: Koudelka_Revised_F2S — Comparing FragPipe and Spectronaut with the LiP-MS direct-DIA quantification mode A, total number of kinase protein targets and common protein kinase targets of staurosporine found using Spectronaut or FragPipe in direct-DIA mode. B, total number of kinase peptide targets and common peptide kinase targets of staurosporine found using Spectronaut or FragPipe in direct-DIA mode. C, receiver operator characteristic (ROC) curves of staurosporine protein interactions, their respective area under the curve (AUC) values, and number of kinases identified (Kinases) measured by DIA LiP-MS with a direct-DIA library and processed by Spectronaut or FragPipe. The dashed line represents a random classifier. The ground truth is represented by the 185 protein kinases detected with the direct-DIA library approach. D, Pearson correlation (r) of the concentrations of drug at which we observed a 50% of the maximum LiP peptide intensities (visualized as - log10 effective concentration - pEC50) extrapolated from the dose-response curves of direct-DIA LiP-MS data quantification with Spectronaut or FragPipe. E, Pearson correlation (r) of the concentrations of drug at which we observed a 50% variation of the maximum LiP peptide intensities (visualized as - log10 effective concentration - pEC50) from LiP-MS dose-response data (pEC50 Direct FragPipe) and pEC50s reported from Kinobeads data (Werner et. al. 2012). [file mmc20.pdf]

**A**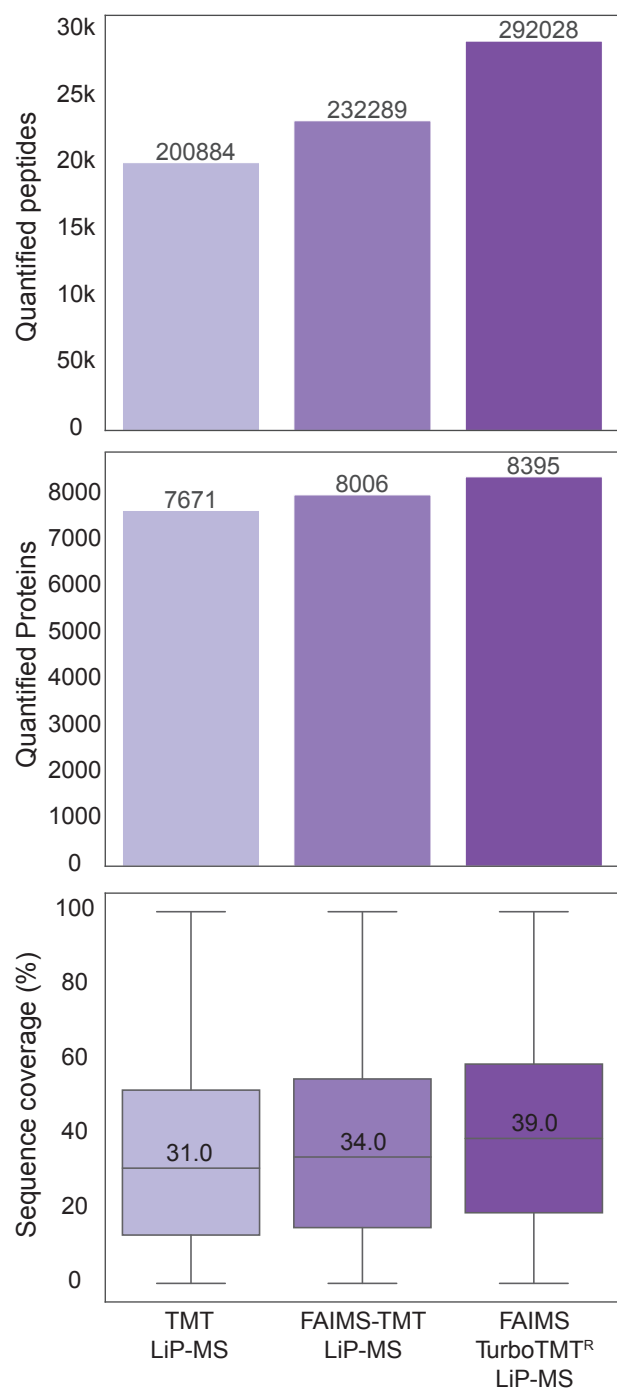**B**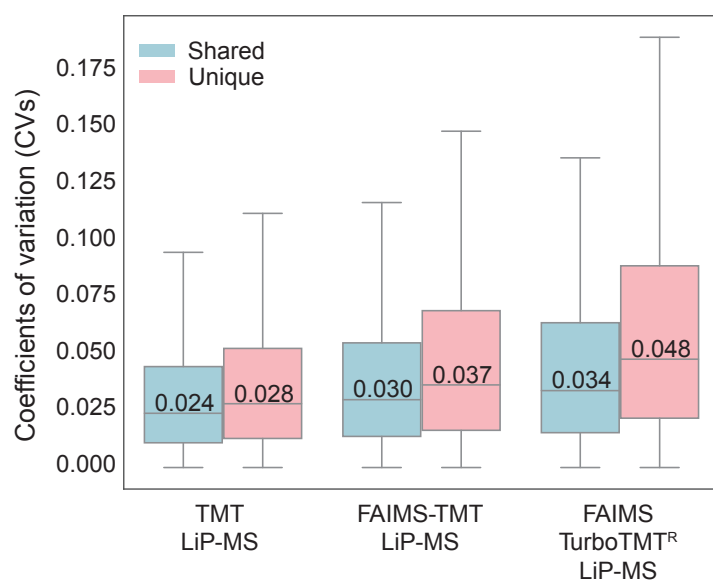**C**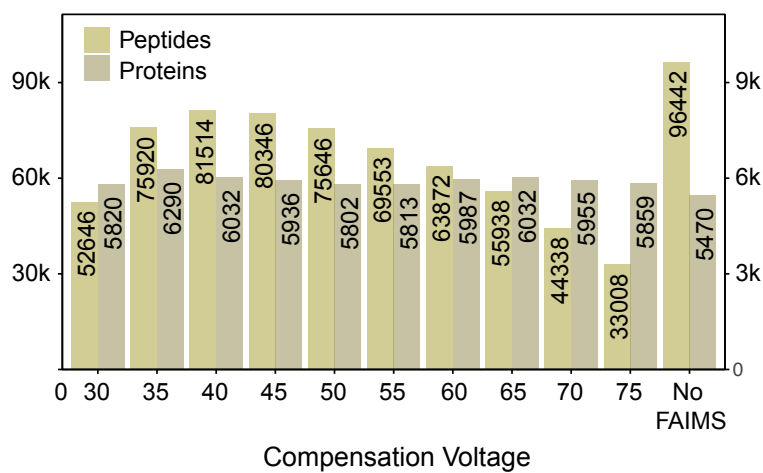**D**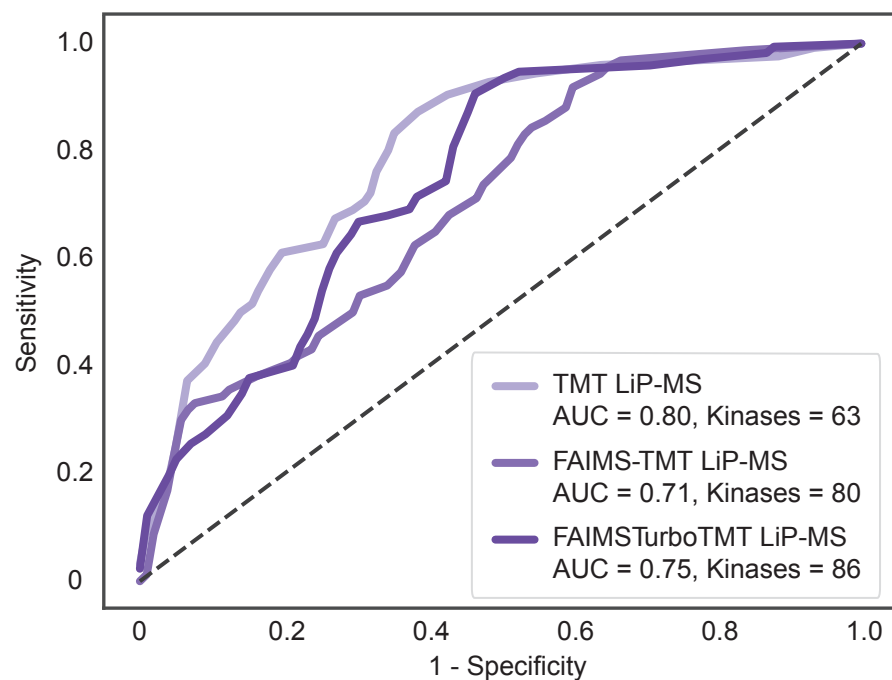

Figure 3S

Supplement: Koudelka_Revised_F3S — Optimization of TMT-based quantification for LiP-MS. A, quantified peptides with the assessed TMT LiP-MS quantification variants: TMT (TMT LiP-MS), TMT with FAIMS (FAIMS-TMT LiP-MS) and TMT with FAIMS and TurboTMT® (FAIMS-TurboTMT® LiP-MS). All options increased both peptide and protein identifications compared to DIA. B, box plots of peptide coefficients of variations (CV). Cyan boxes show overlapping peptides shared among the three different TMT data acquisition settings. Pink box plots illustrate unique peptides quantified exclusively by one specific TMT data acquisition method. The box in each box plot captures the interquartile range with the top and bottom edges representing Q1 and Q3 respectively. The median is the horizontal line within the box. The whiskers length extends to the minima or maxima within 1.5 times the interquartile range below Q1 or above Q3. C, number of peptides and proteins quantified when combining LiP-MS with FAIMS-DIA with different negative compensation voltages. Values correspond to DMSO treated samples (vehicle control) measured with three biological replicates. D, receiver operator characteristic (ROC) curves of staurosporine protein interactions, their respective area under the curve (AUC) values, and number of kinases identified (Kinases) measured with the three assessed TMT LiP-MS quantification variants. The dashed line represents a random classifier. The ground truth is represented by the 185 protein kinases detected by the two quantification methods used here. [file mmc21.pdf]

**A**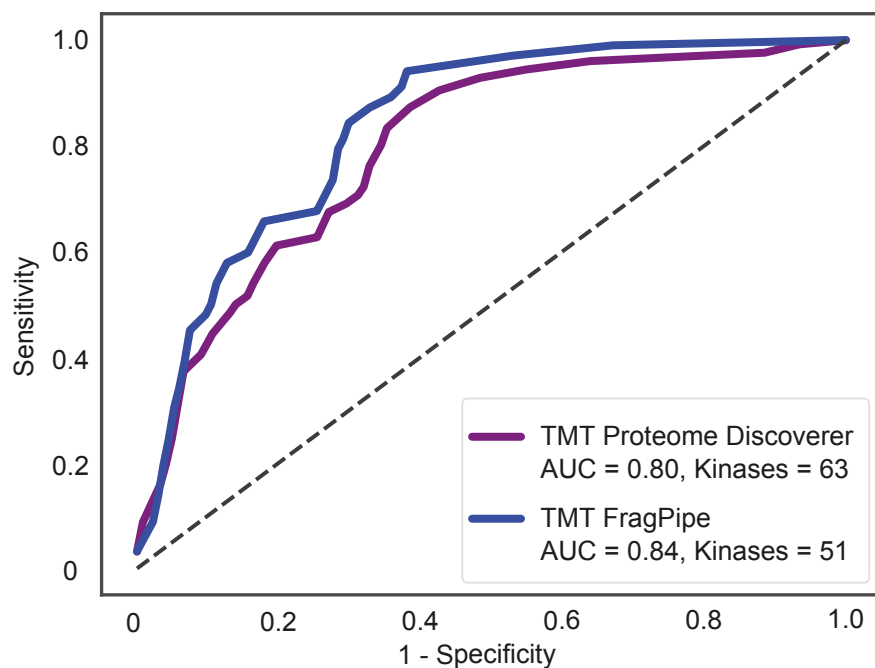**B**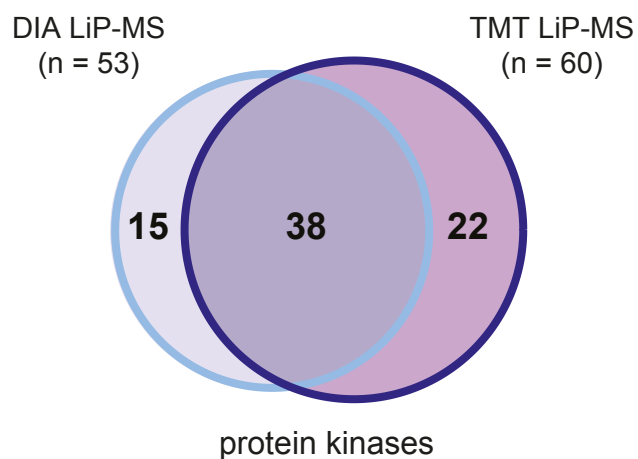**C**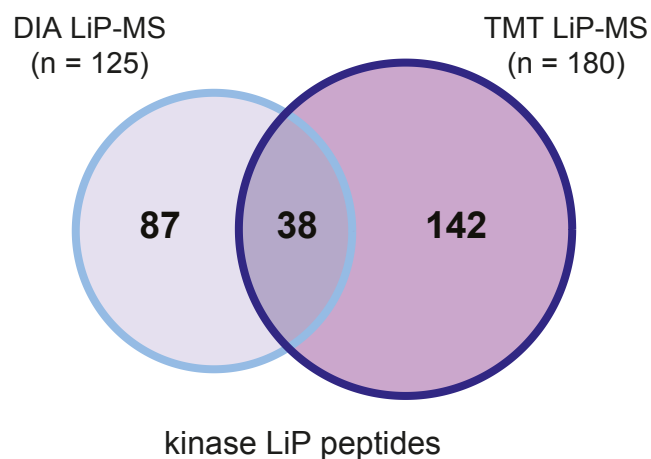**D**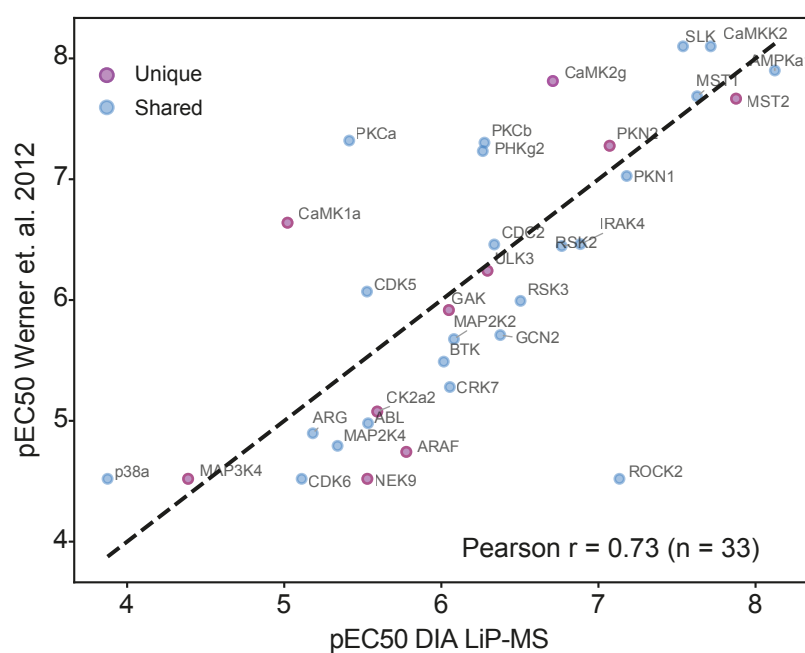**E**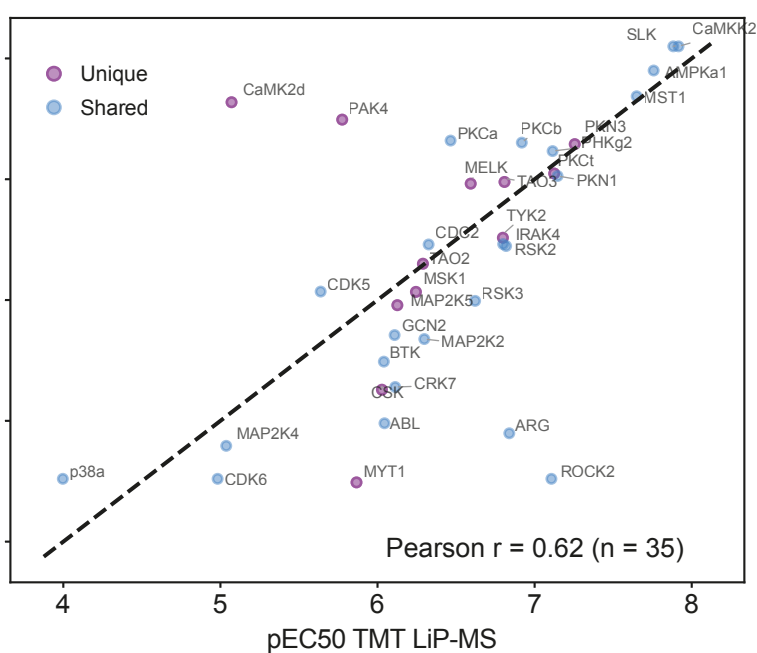

Figure 4S

Supplement: Koudelka_Revised_F4S — Quantitative analysis of drug targets captured by TMT-LiP-MS. A, receiver operator characteristic (ROC) curves of staurosporine protein interactions, their respective area under the curve (AUC) values, and number of kinases identified (Kinases) measured with TMT LiP-MS data analysed with the commercial software Proteome Discoverer or the freeware software FragPipe. The dashed line represents a random classifier. B, total number of kinase protein targets and common protein kinase targets of staurosporine found by DIA LiP-MS or TMT LiP-MS. C, total number of kinase peptide targets and common peptide kinase targets of staurosporine found by DIA LiP-MS or TMT LiP-MS. D, Pearson correlation (r) of the concentrations of drug at which we observed a 50% of the maximum LiP peptide intensities (visualized as - log10 effective concentration - pEC50) from LiP-MS dose-response data (pEC50 DIA LiP-MS) and pEC50s reported from Kinobeads data (Werner et. al. 2012). E, Pearson correlation (r) of the concentrations of drug at which we observed a 50% variation of the maximum LiP peptide intensities (visualized as - log10 effective concentration - pEC50) from LiP-MS dose-response data (pEC50 TMT LiP-MS) and pEC50s reported from Kinobeads data (Werner et. al. 2012). [file mmc22.pdf]
